# Genetic diversity, population structure, and relationships of apricot (*Prunus*) based on restriction site-associated DNA sequencing

**DOI:** 10.1038/s41438-020-0284-6

**Published:** 2020-05-01

**Authors:** Wenwen Li, Liqiang Liu, Yanan Wang, Qiuping Zhang, Guoquan Fan, Shikui Zhang, Yatong Wang, Kang Liao

**Affiliations:** 1 College of Horticulture and Forestry, Xinjiang Agricultural University, Urumqi, Xinjiang 830052 China; 2 Xiongyue National Germplasm Resources Garden of the Liaoning Institute of Pomology, Xiongyue, Shenyang 115009 China; 3 Luntai National Fruit Germplasm Resources Garden of Xinjiang Academy of Agricultural Sciences, Luntai, Xinjiang 841600 China

**Keywords:** Comparative genomics, Genome evolution

## Abstract

Single-nucleotide polymorphisms (SNPs) are the most abundant form of genomic polymorphisms and are widely used in population genetics research. Here, high-throughput sequencing was used to examine the genome-level diversity, population structure, and relationships of apricot, which are important for germplasm conservation and molecular breeding. Restriction site-associated DNA sequencing (RAD-seq) was adopted to sequence 168 *Prunus* spp. accessions distributed in five ecological groups, including 74 accessions of cultivated *Prunus armeniaca* L. and 94 accessions of wild apricots (*P. armeniaca* L. and *Prunus sibirica* L.), which generated 417,961 high-quality SNPs. We used cluster, genetic structure, and principal component analyses to examine the genetic diversities and genetic relationships of the 168 accessions. The Dzhungar-Ili ecological group accessions showed the highest genetic diversity in terms of private allele number, observed heterozygosity, and nucleotide diversity. We speculate that the Central Asian ecological group accessions were domesticated from the Dzhungar-Ili ecological group accessions. The population structure and gene flow of the North China and European ecological group accessions suggested a genetic background of *P. sibirica*. We argue that the two groups should be considered hybrid swarms connected to *P. sibirica* by continuous and extensive gene flow. *P. armeniaca* originated in Northwest China (Ili Valley), subsequently spread throughout Central Asia, and eventually spread to Europe. In addition, selective sweep signatures in *P. armeniaca* during domestication from wild to cultivated apricots, combined with differentially expressed genes, underlie distinct fruit traits, including sugars, aromas, organic acids, and carotenoids. This study provides substantive and valuable genomic resources that will significantly advance apricot improvement and effective utilization.

## Introduction

Apricot is a fruit of temperate and subtropical regions and is distributed worldwide. China, Iran, Pakistan, Uzbekistan, Morocco, Algeria, Ukraine, and the USA are the main producers of apricot^1^. Apricot belongs to section *Armeniaca* (Lam.) Koch, subgenus *Prunophora* Focke, and genus *Prunus* (Rosaceae)^2^. According to different taxonomic systems, apricots are divided into three to twelve species^3,4^. However, six species are recognized by most scholars^3,5,6^: *Prunus armeniaca* L., *Prunus sibirica* L., *Prunus mandshurica* (Maxim.) Skv., *Prunus holosericea* (Batal.) Kost., *Prunus mume* Sieb. et Zucc., and *Prunus brigantina* Vill. Almost all cultivated apricots originated from *P. armeniaca*^7^.

Chinese scholars^8^ divided apricot into six ecological groups based on geographical distribution. The Central Asian ecological group (CAG) accessions are the most abundant, with vigorous tree growth, many slender twigs, and small leaves. These accessions flower late, are self-fruiting, and produce soft, small fruits with a sweet kernel^8^. The European ecological group (EG) accessions are medium-sized trees with thick branches, weak growth, and a short dormancy period, and mostly produce firm fruits through self-fertilization. The flowers, leaves, and fruits of EG accessions are usually large^8^. Accessions in the Dzhungar-Ili ecological group (DZG) are small trees that produce small fruits with a pubescent peel and exhibit strong cold, drought, and waterlogging resistance^8^. The North China ecological group (NCG) accessions are tall trees with vigorous growth. These accessions have intermediate cold and drought resistance and produce fruits via self-fertilization that are large and juicy^8^. The Northeast Asian ecological group (NAG) accessions are small shrubs that have strong cold resistance. These accessions can tolerate temperatures as low as −50 °C in winter, exhibit early leaf fall, and produce small and dry fruits with bitter flesh that are not suitable for consumption^8^. The East China ecological group (ECG) accessions include shrubs or medium-sized trees and tall trees and have strong disease resistance. The seed-setting rate of self-pollinated flowers is very low, the colors of the flowers vary, and the fruits are mostly green^8^. Therefore, the groups exhibit large morphological and physiological differences and wide adaptability to ecological conditions^9^.

According to Vavilov^10^, apricots have three origin centers: China, Central Asia, and the Near East. Most scholars^5,6,11,12^ support the history of ancient apricots in Central Asia and China, regarding these regions as independent centers of domestication. However, the early history of apricot is not completely clear, and the main question of whether the cultivation history in Central Asia is longer or shorter than that in China remains unanswered^13^. De Candolle et al.^14^ argued that apricot cultivation in China dates back to the end of 3000 BC. However, there is no archeological evidence for the beginning of apricot domestication and cultivation. In Central Asia, apricot cultivation was recently introduced in ~1000 to 2000 BC^15^. Many scholars have shown that the genetic diversity of apricots in China is the richest worldwide^16–18^.

The Kashgar, Hotan, and Kuqa oasis areas around the Tarim Basin in the southern part of the Xinjiang Uygur Autonomous Region of China are the main apricot-producing areas and contain the greatest abundance of apricot cultivars. The wild apricot forest in Ili, Xinjiang, China, is a relic of a broad-leaved forest from the late Tertiary community that played a decisive role in the domestication and cultivation of apricot worldwide^18^. It is an important part of the deciduous broad-leaved forest under the mountain coniferous forest and above-mountain grassland in Xinjiang^8^. There is only one mountain between the southern Xinjiang and Ili areas in the northern Tianshan Mountains, and there are several corridors between the northern and southern Tianshan Mountains. Geographically, the apricots cultivated in Xinjiang, southern Tianshan Mountains (CAG), most likely evolved from the spread of wild apricots in the Ili Valley (DZG)^8^.

Crop domestication not only changes economic and agronomic traits but also retains the genetic characteristics that affect the genetic diversity and population structure of domesticated plants^19^. Identifying genetic variations in wild and cultivated populations and within populations can provide insights into the general mechanisms of plant domestication and diversification and guide crop genetic improvement in future breeding programs^20^. There is evidence that domestication can lead to a decrease in the genetic diversity of cultivated crops^21^. The genetic information present before domestication and artificial selection may be retained in wild populations, which are special resources for studying the effects of variation in the apricot genome. However, the natural apricot population in China is strongly influenced by human activities, such as logging and deforestation. Consequently, protective measures must be taken to prevent further declines in wild apricot resources. Information on genetic diversity and population structure is essential for the development of management and conservation approaches. We studied the genetic diversity and population structure of apricot and identified the representative core germplasm. We can preserve the genetic diversity of the whole population to the greatest extent by using the smallest amount of genetic resources, and further improve the protection efficiency of apricot germplasm resources^22^. Developing genomic resources, increasing the knowledge of the apricot gene pool, and obtaining information about genetic diversity and population structure can accelerate biological research and genetic improvement.

The rapid development of high-throughput sequencing technology has provided whole-genome information for nonreference crops. The use of this technology has produced large data sets of short sequences within millions of genomes with deep coverage. These methods, including restriction site-associated DNA sequencing (RAD-seq) and genotyping-by-sequencing (GBS), have been successfully applied in population genomics studies^23–25^. Previous studies used nuclear DNA, mitochondrial DNA, and chloroplast DNA^3,26–28^ sequence fragments to study the genetic diversity of apricots and the relationships between apricot and other related species^29^. Compared with genome-level sequencing, plastid and nuclear gene molecular markers have a lower accuracy and resolution. The limited number of polymorphic loci produced by conventional molecular markers hinders genetic research on apricot.

The main purposes of this study were to evaluate the genetic diversity, population structure, and relationships of apricots in different ecological groups using RAD-seq of the whole genome, clarify the phylogenetic relationships between five ecological groups and provide a theoretical basis for further improving and effectively utilizing apricot germplasm resources.

## Results

### Sequence data quality and processing

**Fig. 1:**
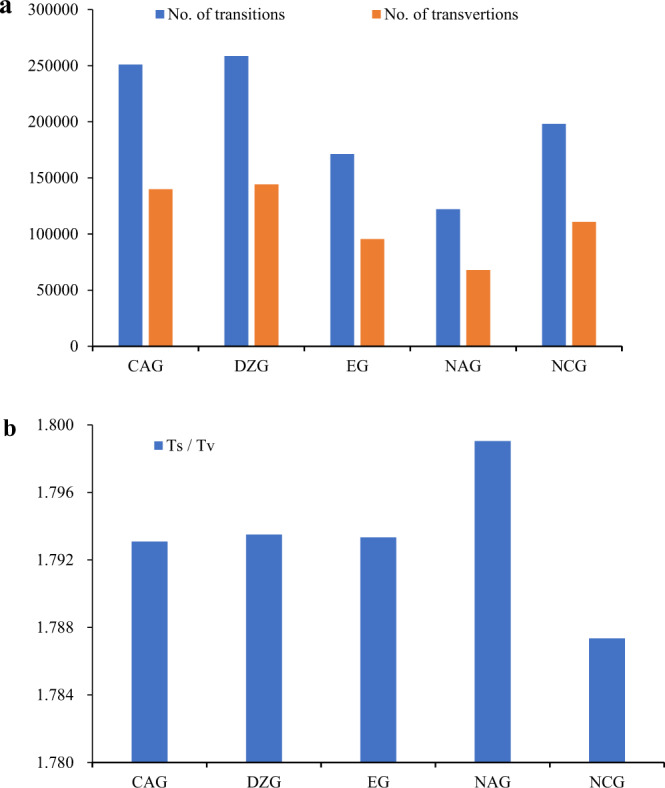
SNP mutation type for the five ecological groups. **a** Number of transitions and transversions for the five ecological groups. **b** transition/transversion rate

A collection of 168 *Prunus* spp. accessions were successfully sequenced using the HiSeq 2000 system (Illumina Inc., USA) producing a total of 192 Gb of raw reads, with an average of 1.14 Gb for each accession. After filtering low-quality reads, a total of 180 Gb of clean data were retained, with an average of 1.07 Gb per accession and an average success rate of 98.44%. The quality of the sequencing data was high (Q20 > 90%, Q30 > 85%), the GC content was stable at 37.33–41.25%, and all samples were free from contamination (Supplementary Data-Table 2). The enzyme capture rate ranged from 92.84 to 98.92%. The mapping rate ranged from 88.7 to 94.37%. A total of 1,475,632 small nucleotides (SNPs) were obtained from the 168 *Prunus* spp. accessions using SAMtools software^30^, and 417,961 SNPs were obtained after filtering, which ensured the accuracy and reliability of subsequent genetic diversity and population structure analyses. At the population level, more than half of the SNPs (with an average of 55.72%) had base transitions, and the transition vs. transversion (ts/tv) ratios for the populations ranged from 1.787 to 1.799. The ts/tv ratios of SNPs in the two wild populations (NAG and DZG) were larger than those in the cultivated populations (Fig. 1; Supplementary Data-Table 3).

### Population phylogenetic relationships

**Fig. 2:**
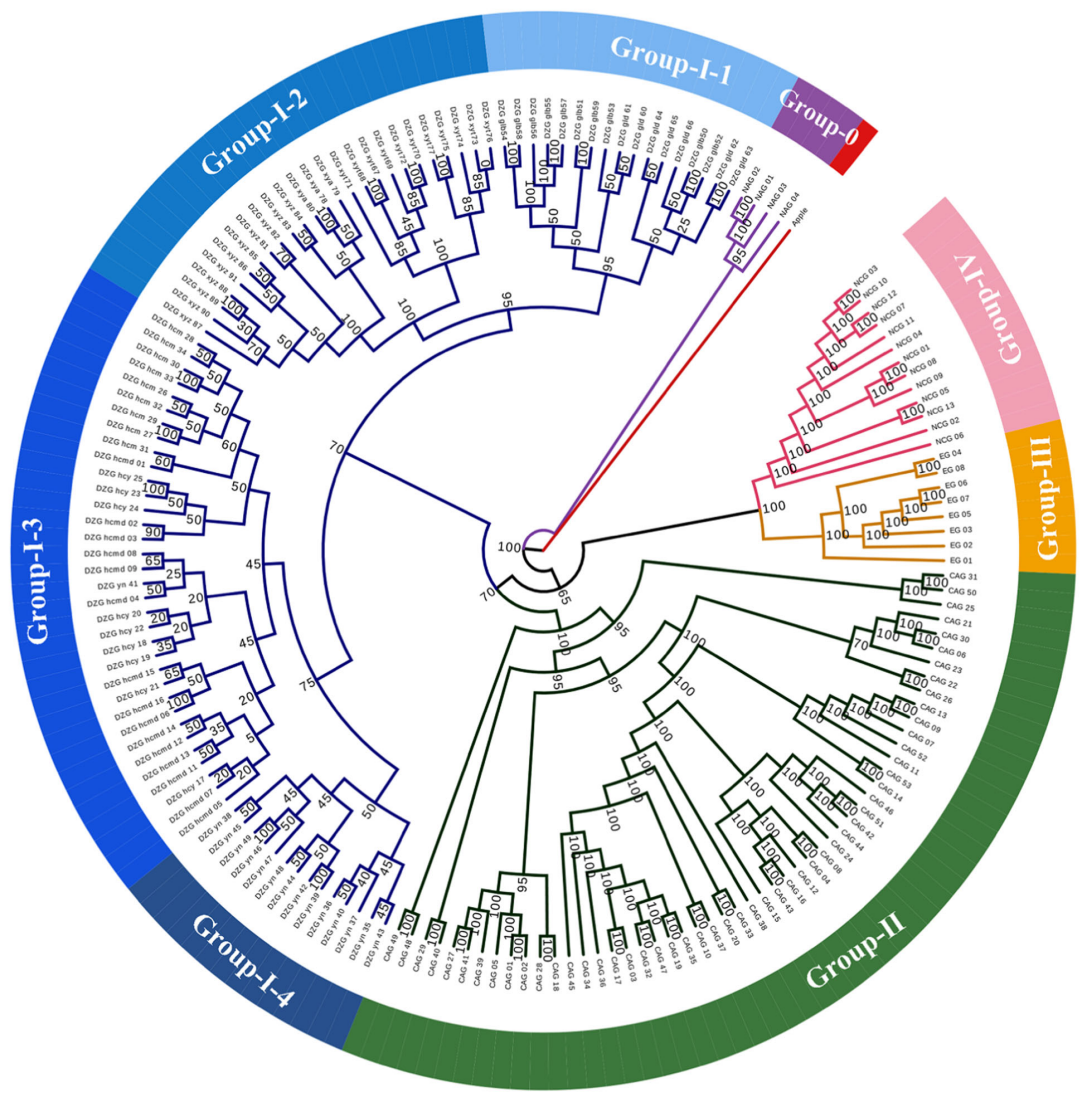
ML tree created for 168 *Prunus* spp. accessions. Red indicates an outgroup (*Malus sieversii*). Group-0 represents Northeast Asian ecological group (wild *P. sibirica*), Group I represents Dzhungar-Ili ecological group (wild *P. armeniaca*), Group II represents Central Asian ecological group (cultivated *P. armeniaca*), Group III represents European ecological group (cultivated *P. armeniaca*), and Group IV represents North China ecological group (cultivated *P. armeniaca*). Node values correspond to bootstrap values

**Fig. 3:**
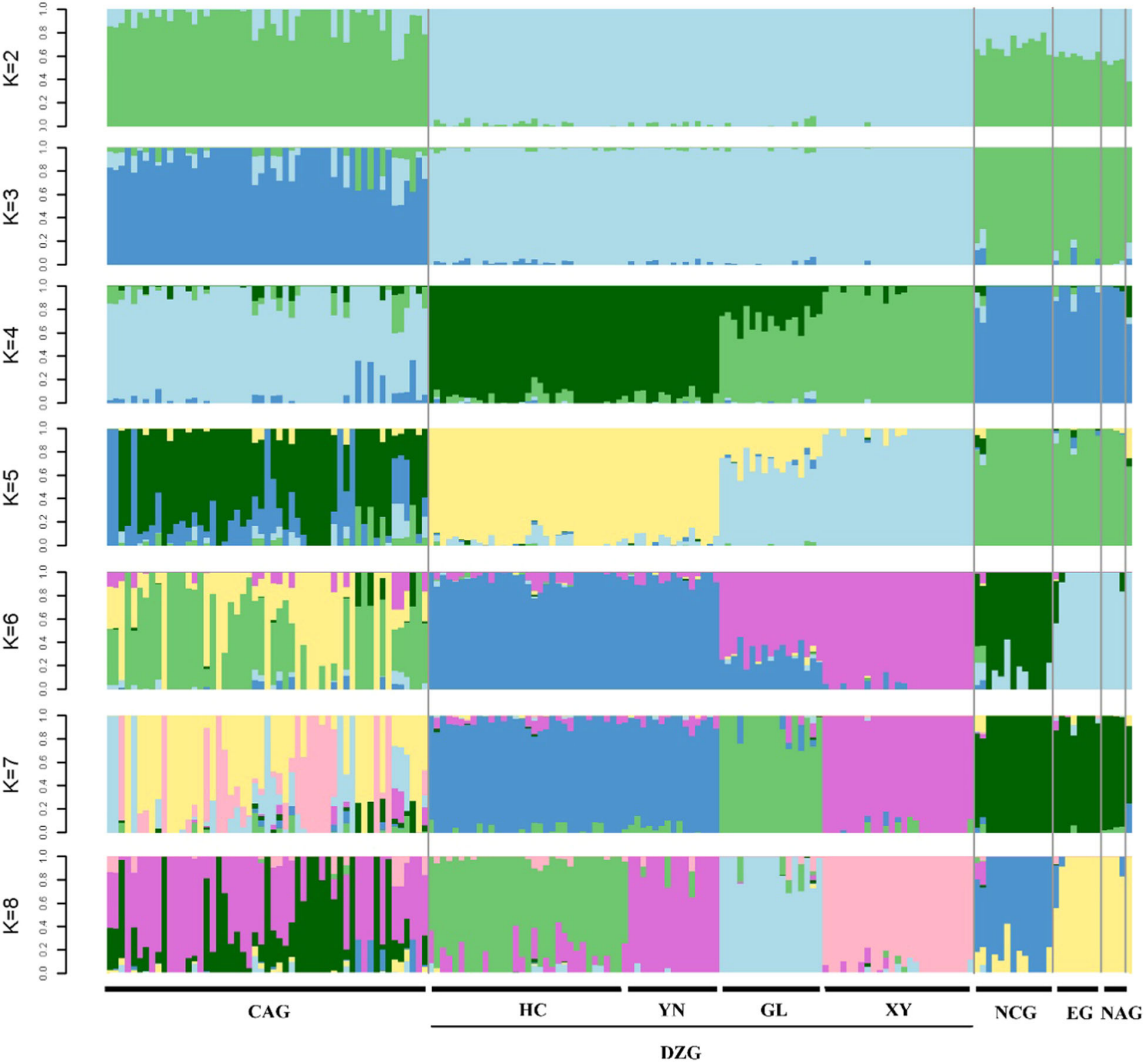
Population structure of 168 accessions of *Prunus* spp. Each column represents an individual, with the length of the different color segments representing the proportion of an ancestor in the individual’s genome. *K* = 2–8 indicates the number of ancestral groups assumed in this study from 2 to 8; The *x*-coordinate indicates the name of the sample, and the order of the sample of the same group was specified together. HC Huocheng county, YN Yining county, GL Gongliu county, XY Xinyuan county

A phylogenetic tree of all 168 accessions was constructed to better understand their relationships (Fig. 2). In the phylogenetic tree, most branches had high bootstrap values, which indicated that the maximum likelihood (ML) tree was highly reliable. The phylogenetic tree divided the collected accessions into five groups. One group included the NAG accessions, located in Northeast China and belonging to *P. sibirica*. These accessions are adapted to and grown in extremely cold regions in China, and are thus considered very hardy. The other four groups belonged to *P. armeniaca*. These groups include (1) the DZG (group I), with four subgroups based on the strong geographical distribution pattern among the branches (Yining County (group I-1), Huocheng County (group I-2), Xinyuan County (group I-3), and Gongliu County (group I-4)) in the Ili region, Xinjiang; (2) the CAG (group II), with apricot cultivars mainly from the Kashgar, Hotan, and Kuqa areas in Xinjiang that could not be clearly distinguished based on their geographical distributions, which may be due to the high genetic diversity and frequent gene exchange generated by selection or introduction, reflecting the complex domestication history of cultivated apricots^18^ (notably, as revealed by the population structure analysis (Fig. 3), the CAG was mixed with the genetic background of DZG; therefore, Xinjiang cultivated apricots originated from Ili wild fruit forests); (3) the EG (group III), with apricot cultivars mainly from North America, Italy, and France; and (4) the NCG (group IV), with apricot cultivars mainly from Northeast China, North China, and Korea.

### Population genetic structure

**Fig. 4:**
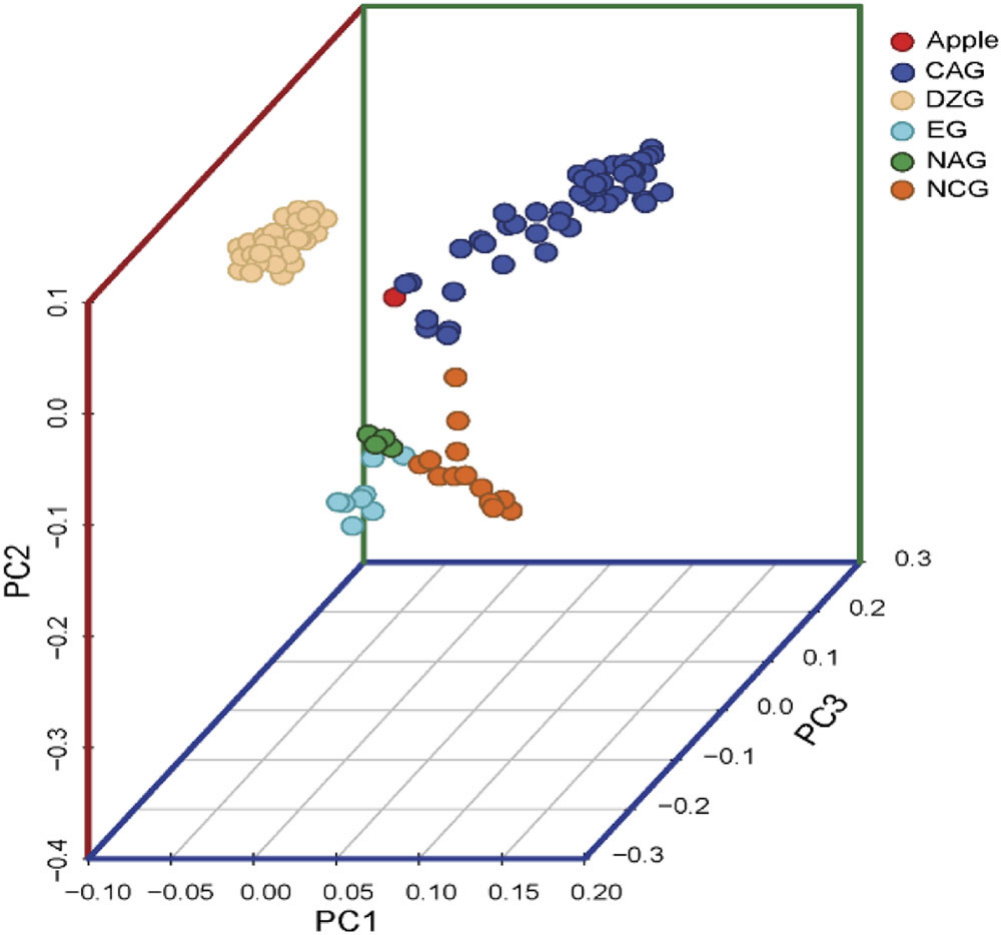
PCA analysis in 168 accessions of *Prunus* spp. Red indicates an outgroup (*Malus sieversii*). The CAG, DZG, EG, NAG, and NAG indicate Central Asian ecological group, Dzhungar-ili ecological group, European ecological group, Northeast Asian ecological group, and North China ecological group

Population genetic structure analysis was conducted based on high-quality SNPs filtered by VCFtools. Based on the ML, *K* = 2–8 ancestral populations were assumed in this study. The optimal population *K* value was determined according to the coefficient of variation (CV), and the optimal value obtained in this study was *K* = 5 (Supplementary Data-Fig. 1). According to Fig. 4, when the *K* value was 5, 168 materials were not assigned to the five groups, which was inconsistent with the results of the phylogenetic tree analysis. Due to divergence in the population structure and phylogenetic tree results, it was necessary to analyze population structure under different *K* values. Therefore, we examined the population structure for *K* values ranging from 2 to 8 (Fig. 3).

When *K* = 3, the CAG and DZG accessions were distinguished, while the EG, NCG, and NAG accessions could not be separated. When *K* = 6, in addition to the CAG and DZG accessions, the NCG and EG accessions could be distinguished from each other. When *K* = 8, the population structure of the CAG, NCG, and EG accessions remained unchanged, and internal differentiation occurred in only the DZG accessions, which were divided into four subgroups: Huocheng, Yining, Gongliu, and Xinyuan. The genetic background of the CAG accessions was complex, but most of the genetic background was found to originate from the DZG accessions, which supports the hypothesis that the cultivated apricots in Xinjiang originated from wild apricots in the Ili region. However, it was interesting to note that the population structure of the EG and NAG accessions changed very little as *K* increased from 2 to 8. The highly polymorphic and diverse properties of the DZG, CAG, and NCG might have influenced the population structure of the EG and NAG. Combined with the phylogenetic tree, these results clearly divided apricot into five groups: CAG, DZG, NCG, EG, and NAG accessions.

### Population genetic relationships

The collection of 168 accessions was analyzed by principal component analysis (PCA) to better understand the relationships between ecological groups (Fig. 4), and the PCA scores were used to evaluate genetic variance. The first three components explained 16.49% of the total genetic variation (8.28, 5.88, and 2.33%, respectively). According to the first three components, the accessions were divided into five groups: the CAG, DZG, NCG, EG, and NAG.

### Gene flow analysis

**Fig. 5:**
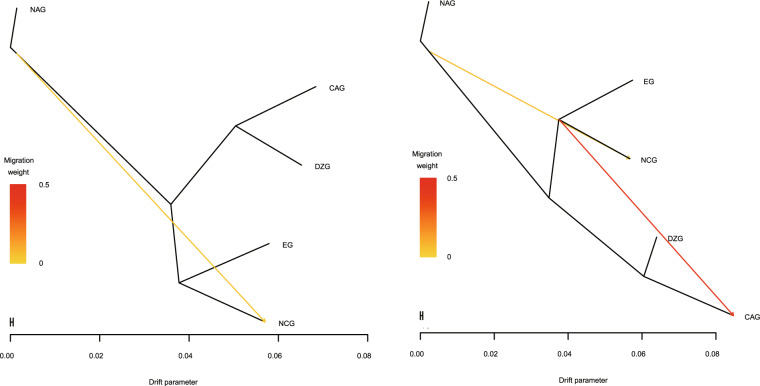
Detection of gene flow between five ecological groups accessions. Lines represent gene flow; arrows indicate the direction of ene flow. The scale bar shows a tenfold average standard error of the entries in the sample covariance matrix. The color bar shows the migration weight: a red color denotes a strong gene flow, while a yellow color denotes a weak gene flow

We analyzed the gene flow between the five ecological groups (Supplementary Data-Fig. 2). Allowing just one migration event (*m* = 1) (Fig. 5a), we observed that gene flow occurred from the NAG to NCG accessions. In terms of geographical location, these two ecological groups were close to each other, which may have caused the germplasm of the NCG accessions to be mixed with that of *P. sibirica*. Allowing two admixture events (*m* = 2) (Fig. 5b), the EG, NCG, and CAG accessions exhibited extensive gene flow, which likely reflects their many shared genomic components due to hybridization in their domestication and breeding histories. This result was consistent with that observed on the basis of population structure (Fig. 3).

### Population genetic diversity

**Fig. 6:**
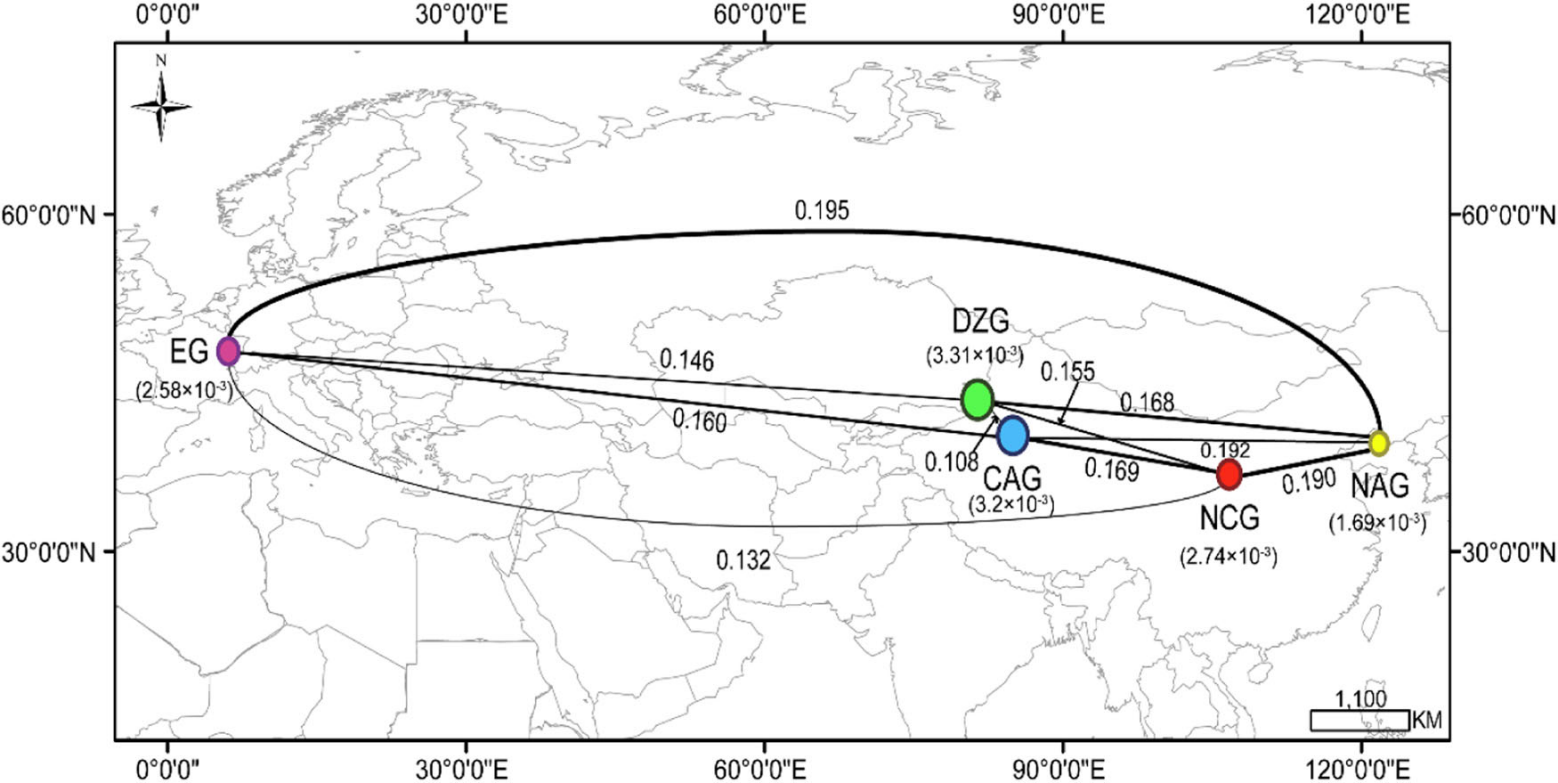
Summary of nucleotide diversity and population divergence across the five ecological groups. Values in parentheses represent measures of nucleotide diversity for the group, and values between pairs indicate population divergence (*F*st). The thickness of the lines is proportional to the value of *F*st. The circle size is proportional to the value of π

The expected heterozygosity (He) of the *P. armeniaca* populations ranged from 0.378 to 0.407; the observed heterozygosity (Ho) of the *P. armeniaca* populations ranged from 0.218 to 0.260; Wright’s F-statistic (*F*_IS_) of the *P. armeniaca* populations ranged from 0.030 to 0.078; and the number of private alleles (A_P_) in the populations of *P. armeniaca* ranged from 11–11,069. The average nucleotide diversity (π) and polymorphism (Waterson’s theta, θw) estimates of the *P. armeniaca* population mutation parameters were 0.0027 and 0.0016 at the genome level, respectively (Table 1, Fig. 6). The mean nucleotide variation of *P. armeniaca* was higher than that of other perennial crops, such as peach (π = 0.0015)^31^ and cassava (π = 0.0026)^32^, but lower than that of date palm (π = 0.0092)^33^.

**Table 1:** The statistical values of genetic diversity within populations from different ecological groups

Taxon	Apricot accessions groups	Private allele number (A_P_)	Effective site	SNP number	Ho	He	*F*_IS_	θw	Tajima’s D
Wild *P. armeniaca*	DZG	11,069	417,961	402,703	0.260	0.378	0.071	2.02E-03	1.035
Cultivated *P. armeniaca*	CAG	3784	417,961	390,892	0.251	0.405	0.078	1.52E-03	0.844
	NCG	202	416,708	308,930	0.223	0.407	0.033	2.20E-03	0.468
	EG	11	411,449	266,769	0.218	0.406	0.030	2.05E-03	0.510
Wild *P. sibirica*	NAG	0	385,166	189,976	0.155	0.391	0.031	1.52E-03	0.220

*Ho* observed heterozygosity, *He* expected heterozygosity, *π* nucleotide diversity, *θw* Watterson’s theta, *F*_IS_ inbreeding coefficient of an individual relative to the population

Further analysis of the DZG accessions (Table 2) showed that the observation heterozygosity (Ho) ranged from 0.373 to 0.383, the expected heterozygosity (He) ranged from 0.256 to 0.268, the inbreeding coefficient (*F*_IS_) ranged from 0.015 to 0.055, and the private allele number (A_P_) ranged from 2026 to 8847. The Ho and A_P_ in Xinyuan County were higher than those in Gongliu, Yining, and Huocheng Counties.

**Table 2:** The statistical values of genetic diversity within groups from wild apricots

Apricot accessions groups	Population	Private allele number (Ap)	Polymorphic loci, %	Ho	He	*F*_IS_
DZG	Xinyuan County	3217	88.1983	0.3828	0.2608	0.0219
	Yining County	2026	85.7091	0.37906	0.26767	0.0152
	Huocheng County	3046	86.4664	0.37277	0.2583	0.037
	Gongliu County	8847	95.0875	0.37768	0.2558	0.0553

Notably, the Ho and π (0.260 and 0.274, respectively) of the DZG accessions were higher than those of the CAG accessions (0.251 and 0.265, respectively), NCG accessions (0.223 and 0.229, respectively) and EG accessions (0.218 and 0.222, respectively) (Fig. 6). Therefore, some of the genetic diversity in apricots was lost during the process of domestication from the DZG to the EG. The genetic diversity index of *P. armeniaca* was larger than that of *P. sibirica*.

Tajima’s D represents intraspecific polymorphism based on locus variation. The Tajima’s D values of the five groups were positive, and significantly different from zero (Table 1). Thus, the null hypothesis of neutral evolution could be rejected. Furthermore, numerous intermediate-frequency alleles were detected in the populations, which may have been caused by a bottleneck effect, population structure, or balancing selection.

### Genetic differentiation and AMOVA

The genetic differentiation index (*F*st) between populations ranged from 0.1080 to 0.1946 (Fig. 6), and the degree of differentiation among populations was low. The *F*st values of the CAG and DZG accessions were the smallest among all the groups analyzed (Fig. 6), indicating that the genetic differences within populations were greater than those between populations and the occurrence of gene flow between populations^34,35^. The *F*st values of the EG and NAG accessions were the largest among all the groups analyzed (Fig. 6), which may have been due to geographical isolation, low gene flow between populations, and large genetic differences^34,35^.

Assessment of genetic differences amDNA was extracted from young leavesong the five geographical groups by analysis of molecular variance (AMOVA) revealed that 12.02% of the genetic variation occurred between populations and 87.98% occurred within populations (Table 3). Although the variation among the five geographical groups of accessions mainly occurred within populations, the differences between populations were also large.

**Table 3:** Results of the analyses of molecular variance from five ecological groups

Source of variation	Degree of freedom	Sum of squares	Variance components	Percentage of variation
Among populations	5	82466.52	340.03 Va	12.02
Within populations	332	826059.33	2488.13 Vb	87.98
Total	337	908525.85	2828.16	

### Scans for selective sweeps

**Fig. 7:**
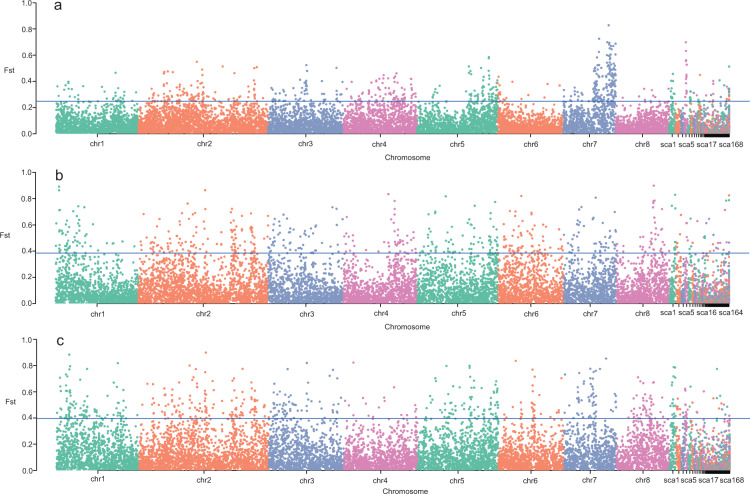
Distribution of *F*st values of selective sweeps of *P. armeniaca* during domestication from wild to cultivated apricots. **a** Distribution of *F*st values of selective sweeps in DZG and CAG accessions during domestication. **b** Distribution of *F*st values of selective sweeps in DZG and EG accessions during domestication. **c** Distribution of *F*st values of selective sweeps in DZG and NCG accessions during domestication. The horizontal line indicates the threshold of *F*st (5%)

Separate selective sweeps driven by artificial selection were detected in the CAG, EG, and NCG accessions. For CAG accessions, selective sweep signatures were detected for 0.27 Mb of the genome sequence, containing 53 putative genes (Fig. 7a; Supplementary Data-Table 5). For EG accessions, selective sweep signatures were detected for 1.22 Mb of the genome sequence, containing 278 putative genes (Fig. 7b; Supplementary Data-Table 7). For NCG accessions, selective sweep signatures were detected for 1.19 Mb of the genome sequence, containing 290 putative genes (Fig. 7c; Supplementary Data-Table 9).

One carotenoid biosynthesis gene, one cellulose biosynthesis gene, two serine and threonine metabolism genes, and two GTPase regulatory genes were identified in the selective sweep regions of the CAG accessions (Supplementary Data-Table 4). Eight serine and threonine metabolism genes, three ethylene biosynthesis genes, two aromatic metabolism genes, two organic acid metabolism genes, one high-affinity transport system gene, two stress tolerance-related genes, one pectin degradation gene, one pathogen-related gene, and one salicylic acid biosynthesis gene were detected in the EG accessions (Supplementary Data-Table 6). One auxin-related gene, thirteen serine and threonine metabolism genes, five GTPase regulatory genes, five ethylene biosynthesis genes, one organic acid metabolism gene, one stress tolerance-related gene, one pathogen-related gene, one salicylic acid biosynthesis gene, three sugar metabolism genes, one auxin-related gene, one alanine metabolism gene, one aspartate metabolism gene, one glutamate metabolism gene, and one glucuronoxylan biosynthesis gene were detected in the NCG accessions (Supplementary Data-Table 8).

The candidate genes identified in selective sweeps of *P. armeniaca* during domestication from wild to cultivated apricots differ greatly in function, indicating that different ecological groups of *P. armeniaca* adapted to different selection pressures for desired agronomic characteristics in cultivated apricot. Thus, the domestication of cultivated apricot was a process of differential selection.

## Discussion

Genomic data provide a novel perspective for studying the genetic diversity and domestication of apricot. In this study, we collected 168 accessions from five geographical groups and performed whole-genome restriction enzyme digestion to quickly obtain highly accurate variation information based on markers. The sample sizes averaged 1.14 Gb, ensuring the accuracy of the population genetic analysis. We used RAD-seq to detect 417,961 SNPs, which exceeded the numbers of amplified fragment length polymorphism (AFLP) and random amplified polymorphic DNA (RAPD) markers.

Substitution between purine and purine or between pyrimidine and pyrimidine is called transition, and substitution between purine and pyrimidine is called transversion. Point mutations are common in organisms and cause a wide variety of phenotypic changes. This nucleotide mutation pattern is also observed in other plants, such as peanut^36^, maize^37^, *Amorphophallus paeoniifolius*^38^, and *Arabidopsis*^39^. Due to the biased mutation process in the plant genome, the predicted number of transitions is much larger than the predicted number of transversions^38^. Consistent with these predictions, we found that the base transition values were higher than the base transversion values and that the ts/tv ratios of the five geographical groups ranged from 1.787 to 1.799 (Fig. 1), indicating strong transition bias. Further analysis showed that the ts/tv ratio of the cultivated population was lower than that of the wild population, which may have been due to long-term, strict artificial selection, and the loss of evolutionary potential.

The genetic diversity index of the cultivated population was much lower than that of the wild population (*P. armeniaca*) (Table 1, Fig. 6), and the genetic diversity of the EG accessions was lower than that of the other groups of accessions. The A_P_ was most significant in the wild population (DZG), measuring 11,069, indicating a large pool of genetic variation, which could guide the genetic improvement of crops in future breeding programs. In contrast, A_P_ values of only 11–3784 (Table 1) were found in the domesticated population (*P. armeniaca*). These differences may be due to inbreeding or continuous directional selection during the domestication process, which narrows the genetic basis of germplasm, reduces genetic diversity, and increases the possibility of genetic drift during the domestication process. Similar results were obtained by Li et al.^26^.

According to Vavilov^10^, China and Central Asia were the two main centers of apricot domestication. Most cultivated apricots belong to *P. armeniaca*. Among the four main ecological groups of *P. armeniaca* species, Kostina^40^ argued that DZG accessions are the most primitive and that CAG accessions have the longest cultivation history. The cultivation of EG accessions has a relatively recent history, being traced back to ~2000 years ago^16^. In Central Asia, local cultivars are most likely to have originated from wild apricots, moving southward from the Kazakhstan–China border (Dzhungar-Ili) to Kashmir and westward into the mountains of Afghanistan^16^. In our study, 168 accessions were divided into five ecological groups based on population structure, PCA and ML tree analysis. The CAG and DZG accessions were tightly clustered, indicating that the DZG (wild apricot) may be the ancestor of the CAG. Zhebentyayeva et al.^18^ and Li et al.^26^ reached similar conclusions. The genetic background of the CAG was most similar to that of the DZG (Fig. 4). The genetic diversity indexes (Ho, A_P_, and *π*) of the DZG accessions were higher than those of accessions in the other ecological groups (Table 1, Fig. 6), which showed the lowest genetic differentiation for CAG accessions (Fig. 6), further supporting the CAG accessions as having originated from the DZG accessions. We speculate that the DZG accessions are the common ancestors of the CAG, NCG, and EG accessions. *P. armeniaca* originated in Northwest China (Ili Valley), subsequently dispersed throughout Central Asia, and eventually spread to Europe. These findings are reasonable from a historical perspective, as there was extensive cultural contact along the Silk Road from 207 BCE to 220 CE^41^. Therefore, historical and commercial influences may have contributed to the development of this unique species of cultivated apricot.

Further analysis of the DZG (Fig. 2) clustered the Xinyuan County and Gongliu County accessions into one group and the Yining County and Huocheng County accessions into another group, providing further geographical support for the relationships among these accessions. According to the genetic diversity indexes (Table 2), the Xinyuan County accessions were the most diverse. He et al.^42^ also found that wild apricots in the Yili Valley maintained a high level of diversity and that genetic variation mainly occurred within populations.

The population structure and gene flow analyses of the NCG and EG accessions suggested a genetic background of *P. sibirica*. We argue that the two groups should be considered hybrid swarms connected to *P. sibirica* by continuous and extensive gene flow.

Human intervention via the artificial selection of favorable phenotypic traits to enhance production and improve desirable agronomic traits can both reduce the levels of genetic variability and skew allele frequencies^43^. The different genes identified in selective sweeps of CAG, EG, and NCG apricots were found to be enriched in biological processes, including the metabolism of sugars, aromas, organic acids, and carotenoids. These genes may have been involved in different domestication pathways, leading to differences in traits among apricot ecological groups. Some sugar-related genes have been found in selective sweeps, suggesting a preference for sweet fruit during domestication. For the NCG accessions (Supplementary Data-Table 8), a total of three sugar-related genes were identified in the selected regions, including two genes that encode enzymes (vacuolar invertase and neutral invertase). Two organic acid genes were identified in the EG accessions (Supplementary Data-Table 6), and only one was identified in the NCG accessions, indicating that the dominant acid components of apricot differ among ecological groups. Fewer genes involved in carotenoid biosynthesis were identified in selective sweeps. One carotenoid biosynthesis gene was identified in the CAG accessions (Supplementary Data-Table 4), indicating a preference for a certain color during domestication. For volatile compounds in apricot fruit, two genes related to aromatic metabolism (fatty acid desaturase and cytochrome P450) were identified in selective sweeps of the EG accessions (Supplementary Data-Table 6), indicating that aroma in these groups of apricots is regulated by different genes in the metabolic pathway.

## Conclusion

In this study, we report genomic information for 168 wild and cultivated apricot accessions. Our findings provide insights into the dissemination of *P. armeniaca*. Selective sweep signatures in *P. armeniaca* during domestication from wild to cultivated apricots, combined with differentially expressed genes, underlie distinct fruit traits, including sugars, aromas, organic acids, and carotenoids. Population structure analysis provided new evidence for the mixed genetic background of apricot in different ecological groups. Our study provides abundant genomic resources for wild and cultivated apricots and makes important contributions to the genetic improvement and molecular breeding of apricot.

## Materials and methods

### Plant material

A diverse collection of 168 *Prunus* spp. accessions encompassing five ecological groups, namely, the CAG (53 domesticated landraces or cultivars), NCG (13 cultivars), EG (8 cultivars), DZG (90 cultivars), and NAG (4 wild accessions), were considered for RAD-seq.

Seventy-four of the accessions were provided by the Luntai National Fruit Germplasm Resources Garden of the Xinjiang Academy of Agricultural Sciences, the Yingjisha County Apricot National Forest Germplasm Bank, and the Xiongyue National Germplasm Resources Garden of the Liaoning Institute of Pomology. Ninety wild apricots (*P. armeniaca*) were collected from the natural distribution areas of wild fruit forests in Xinyuan County, Gongliu County, Yining County and Huocheng County in Ili, Xinjiang. Four wild apricots (*P. sibirica*) were provided by the Xiongyue National Germplasm Resources Garden of the Liaoning Institute of Pomology (Supplementary Data-Table 1).

### DNA extraction, library preparation, and sequencing

DNA was extracted from young leaves using a Plant Genomic DNA Kit (Tiangen, Beijing, China)^44^. The purity and integrity of DNA were analyzed by 1% agarose gel electrophoresis and a NanoDrop® spectrophotometer (ND-1000, Thermo Fisher Scientific, USA). The DNA concentration was accurately quantified with a Qubit^TM^ 2.0 fluorometer (Invitrogen, Carlsbad, CA, USA), after which the DNA was stored at −20 °C^34^. RAD library preparation and sequencing were conducted by Novogene Bioinformatics Technology Co. Ltd., Beijing. The DNA concentration used for library preparation was 75 ng/μl, and the qualified DNA was digested by the *EcoRI* (GAATTC) restriction enzyme. Each sample was physically randomly fragmented by the Covaris S220 system (Covaris, Woburn, MA, USA), and then subjected to barcode ligation, gel fragment selection, adapter connection and PCR fragment amplification, and 300–500 bp sequences were recovered^38^. Paired-end sequencing was performed on the Illumina HiSeq 2000 platform (Illumina, San Diego, CA, USA), and each sample generated ~2.32 Gb of raw data. The clean sequencing data have been submitted to the NCBI Short Read Achieve with accession number PRJNA592274.

### Mapping and SNP calling

Qualified sequencing data were aligned to the CAG 09 reference genome (*P. armeniaca* cv. “Cuijianali”; NCBI accession number PRJNA592274) by Burrows-Wheeler Aligner (version 0.7.8-r455)^45^. We used SAMtools software^30^ to detect SNPs in the population. The final product was converted to variant call format (VCF).

### Sequencing and SNP-calling statistics

To obtain high-quality data, the raw data were sequenced, compared, assessed for mutations, screened, and analyzed. After the reads were successfully aligned to the reference genome through quality control, SAMtools^30^ software was used to calculate read lengths. The ts/tv ratio was obtained with BCFtools (version 1.8)^30^.

### Population structure analysis

After filtering 1,475,632 SNPs (filter conditions: depth < 5, proportion missing > 0.5, and minor allele frequency (maf) < 0.05), 417,961 SNPs were obtained for PCA, population structure analysis and phylogenetic tree construction.

To clarify the genetic relationships among the five ecological groups, phylogenetic trees were constructed. We used the published RAD-seq data of *M. sieversii*^46^ (NCBI accession number SRX2934932) as an outgroup. TreeBeST (version 1.9.2)^47^ was used to calculate the distance matrix, RAxML (version 8.2.12)^48^ was used to construct the ML phylogenetic tree, and 1000 bootstrap replicates were used.

Population structure was analyzed by the frappe program^49^ by setting the number of genetic groups from 2 to 8 and using 1000 bootstrap replicates. To determine the optimal group structure classification, the optimal group number was identified as that with the minimum CV^50^. Group structure was analyzed by PCA using GCTA^51^ software, and the significance level of feature vectors was determined by using a Tracy–Widom test. Genome-wide complex trait analysis (GCTA) software integrates the layered correction method of Eigenstart^52,53^, which can clearly distinguish cases and controls.

### Gene flow analysis

Based on the SNPs, we used TreeMix (version 1.13) to evaluate the gene flow between the five ecological groups with the “-se-bootstrap-k 1000 -m” setting, where the number (-m) varied from 1 to 3.

### Population genetic diversity and differentiation analysis

The genetic diversity indexes included A_P_, Ho, He, π^43^, θw^54^, *F*_IS_, and Tajima’s D^43^. Sliding windows with a 5 kb step size and 10 kb size were used to calculate Ho, He, π, θw, and Tajima’s D. For each window, these values were run using the Bio::PopGen package with a custom Perl script^55^. Paired *F*st values^56^ were calculated in the same window to measure the difference between populations. *F*_IS_^29^ was calculated using the POPULATION program in the STACKS pipeline. The statistical values were all calculated using VCFtools (version 0.1.14)^57^.

Using AMOVA in Arlequin (version 3.5.1)^58^, different genetic structures were defined and statistically tested by classifying and dividing the populations at different levels.

### Identification of selective sweeps

Qualified sequencing data were aligned to the *P. mume* L^59^. reference genome to identify selective sweeps in the 168 *Prunus* spp. accessions distributed in five ecological groups. The results were plotted using the ggplot2 package in R software.

## Supplementary information

Table S1,Table S2,Table S3,Table S4,Table S5,Table S6,Table S8,Fig. 1,Fig. 2
