# Supplementary material for: Genetic diversity, population structure, and relationships of apricot (Prunus) based on restriction site-associated DNA sequencing
Source: Hortic Res. 2020 May 1;7:69. doi: 10.1038/s41438-020-0284-6 (PMC7192913; doi:10.1038/s41438-020-0284-6)
Supplement: Supplementary file 1 — Table S1,Table S2,Table S3,Table S4,Table S5,Table S6,Table S8,Fig. 1,Fig. 2 [file 41438_2020_284_MOESM1_ESM.docx]

**Table S1 Voucher information for taxa used in this study.**

| Accessions | Voucher localities | Voucher data | Collector’s Name | Vocher number | Ecological group | Geographic location |
| --- | --- | --- | --- | --- | --- | --- |
| Cultivated |  |  |  |  |  |  |
| *P. armeniaca* L. cv. 'tuohuti' | China, Xinjiang Uygur Autonomous Region, Luntai County  Xinjiang Uygur Autonomous Region, Luntai County | 28 April 2018 | Wenwen Li, Yanan Wang | CAG 25 | CAG | China (Xinjiang) |
| *P. armeniaca* L. cv. 'danxing' | China, Xinjiang Uygur Autonomous Region, Luntai County | 28 April 2018 | Wenwen Li, Yanan Wang | CAG 50 | CAG | China (Xinjiang) |
| *P. armeniaca* L. cv. 'maolaxiao' | China, Xinjiang Uygur Autonomous Region, Luntai County | 28 April 2018 | Wenwen Li, Yanan Wang | CAG 31 | CAG | China (Xinjiang) |
| *P. armeniaca* L. cv. 'dashushanggan' | China, Xinjiang Uygur Autonomous Region, Luntai County | 28 April 2018 | Wenwen Li, Yanan Wang | CAG 48 | CAG | China (Xinjiang) |
| *P. armeniaca* L. cv. 'xiaoshushanggan' | China, Xinjiang Uygur Autonomous Region, Luntai County | 28 April 2018 | Wenwen Li, Yanan Wang | CAG 49 | CAG | China (Xinjiang) |
| *P. armeniaca* L. cv. 'daguohuanna' | China, Xinjiang Uygur Autonomous Region, Luntai County | 28 April 2018 | Wenwen Li, Yanan Wang | CAG 01 | CAG | China (Xinjiang) |
| *P. armeniaca* L. cv. 'kalahuanna' | China, Xinjiang Uygur Autonomous Region, Luntai County | 28 April 2018 | Wenwen Li, Yanan Wang | CAG 02 | CAG | China (Xinjiang) |
| *P. armeniaca* L. cv. 'kuikepiman' | China, Xinjiang Uygur Autonomous Region, Luntai County | 28 April 2018 | Wenwen Li, Yanan Wang | CAG 05 | CAG | China (Xinjiang) |
| *P. armeniaca* L. cv. 'hexieke' | China, Xinjiang Uygur Autonomous Region, Luntai County | 28 April 2018 | Wenwen Li, Yanan Wang | CAG 18 | CAG | China (Xinjiang) |
| *P. armeniaca* L. cv. 'mugeyageleke' | China, Xinjiang Uygur Autonomous Region, Yingjisha County | 26 April 2018 | Kang Liao, Wenwen Li, Yanan Wang | CAG 28 | CAG | China (Xinjiang) |
| *P. armeniaca* L. cv. 'akenawati' | China, Xinjiang Uygur Autonomous Region, Yingjisha County | 26 April 2018 | Kang Liao, Wenwen Li, Yanan Wang | CAG 39 | CAG | China (Xinjiang) |
| *P. armeniaca* L. cv. 'ximixi' | China, Xinjiang Uygur Autonomous Region, Luntai County | 28 April 2018 | Wenwen Li, Yanan Wang | CAG 41 | CAG | China (Xinjiang) |
| *P. armeniaca* L. cv. 'suogeyageleke' | China, Xinjiang Uygur Autonomous Region, Yingjisha County | 26 April 2018 | Kang Liao, Wenwen Li, Yanan Wang | CAG 27 | CAG | China (Xinjiang) |
| *P. armeniaca* L. cv. 'akeyulvke' | China, Xinjiang Uygur Autonomous Region, Luntai County | 28 April 2018 | Wenwen Li, Yanan Wang | CAG 11 | CAG | China (Xinjiang) |
| *P. armeniaca* L. cv. 'dayoujia' | China, Xinjiang Uygur Autonomous Region, Luntai County | 28 April 2018 | Wenwen Li, Yanan Wang | CAG 14 | CAG | China (Xinjiang) |
| *P. armeniaca* L. cv. 'huangrouyouxing' | China, Xinjiang Uygur Autonomous Region, Luntai County | 28 April 2018 | Wenwen Li, Yanan Wang | CAG 53 | CAG | China (Xinjiang) |
| *P. armeniaca* L. cv. 'luopuhongdeke' | China, Xinjiang Uygur Autonomous Region, Luntai County | 28 April 2018 | Wenwen Li, Yanan Wang | CAG 07 | CAG | China (Xinjiang) |
| *P. armeniaca* L. cv. 'cuijianali' | China, Xinjiang Uygur Autonomous Region, Luntai County | 28 April 2018 | Wenwen Li, Yanan Wang | CAG 09 | CAG | China (Xinjiang) |
| *P. armeniaca* L. cv. 'pinaizi' | China, Xinjiang Uygur Autonomous Region, Luntai County | 28 April 2018 | Wenwen Li, Yanan Wang | CAG 52 | CAG | China (Xinjiang) |
| *P. armeniaca* L. cv. 'baiyouxing' | China, Xinjiang Uygur Autonomous Region, Luntai County | 28 April 2018 | Wenwen Li, Yanan Wang | CAG 13 | CAG | China (Xinjiang) |
| *P. armeniaca* L. cv. 'akeyageleke' | China, Xinjiang Uygur Autonomous Region, Luntai County | 28 April 2018 | Wenwen Li, Yanan Wang | CAG 06 | CAG | China (Xinjiang) |
| *P. armeniaca* L. cv. 'zaodayouxing' | China, Xinjiang Uygur Autonomous Region, Luntai County | 28 April 2018 | Wenwen Li, Yanan Wang | CAG 21 | CAG | China (Xinjiang) |
| *P. armeniaca* L. cv. 'qiaolepan' | China, Xinjiang Uygur Autonomous Region, Yingjisha County | 26 April 2018 | Kang Liao, Wenwen Li, Yanan Wang | CAG 22 | CAG | China (Xinjiang) |
| *P. armeniaca* L. cv. 'kezilang' | China, Xinjiang Uygur Autonomous Region, Luntai County | 28 April 2018 | Wenwen Li, Yanan Wang | CAG 23 | CAG | China (Xinjiang) |
| *P. armeniaca* L. cv. 'mayisaimu' | China, Xinjiang Uygur Autonomous Region, Yingjisha County | 26 April 2018 | Kang Liao, Wenwen Li, Yanan Wang | CAG 26 | CAG | China (Xinjiang) |
| *P. armeniaca* L. cv. 'akeayi' | China, Xinjiang Uygur Autonomous Region, Luntai County | 28 April 2018 | Wenwen Li, Yanan Wang | CAG 30 | CAG | China (Xinjiang) |
| *P. armeniaca* L. cv. 'dabaiyouxing' | China, Xinjiang Uygur Autonomous Region, Luntai County | 28 April 2018 | Wenwen Li, Yanan Wang | CAG 42 | CAG | China (Xinjiang) |
| *P. armeniaca* L. cv. 'youmaoxiaowuyuexing' | China, Xinjiang Uygur Autonomous Region, Luntai County | 28 April 2018 | Wenwen Li, Yanan Wang | CAG 46 | CAG | China (Xinjiang) |
| *P. armeniaca* L. cv. 'kabakeyulvke' | China, Xinjiang Uygur Autonomous Region, Luntai County | 28 April 2018 | Wenwen Li, Yanan Wang | CAG 51 | CAG | China (Xinjiang) |
| *P. armeniaca* L. cv. 'jianali' | China, Xinjiang Uygur Autonomous Region, Luntai County | 28 April 2018 | Wenwen Li, Yanan Wang | CAG 08 | CAG | China (Xinjiang) |
| *P. armeniaca* L. cv. 'wangenmao 2' | China, Xinjiang Uygur Autonomous Region, Yingjisha County | 26 April 2018 | Kang Liao, Wenwen Li, Yanan Wang | CAG 24 | CAG | China (Xinjiang) |
| *P. armeniaca* L. cv. 'dawuyuexing' | China, Xinjiang Uygur Autonomous Region, Luntai County | 28 April 2018 | Wenwen Li, Yanan Wang | CAG 44 | CAG | China (Xinjiang) |
| *P. armeniaca* L. cv. 'luopu 1' | China, Xinjiang Uygur Autonomous Region, Luntai County | 28 April 2018 | Wenwen Li, Yanan Wang | CAG 04 | CAG | China (Xinjiang) |
| *P. armeniaca* L. cv. 'gumuxing' | China, Xinjiang Uygur Autonomous Region, Luntai County | 28 April 2018 | Wenwen Li, Yanan Wang | CAG 12 | CAG | China (Xinjiang) |
| *P. armeniaca* L. cv. 'cuheiyexing' | China, Xinjiang Uygur Autonomous Region, Luntai County | 28 April 2018 | Wenwen Li, Yanan Wang | CAG 16 | CAG | China (Xinjiang) |
| *P. armeniaca* L. cv. 'yechengheiyexing' | China, Xinjiang Uygur Autonomous Region, Luntai County | 28 April 2018 | Wenwen Li, Yanan Wang | CAG 15 | CAG | China (Xinjiang) |
| *P. armeniaca* L. cv. 'keziximixi' | China, Xinjiang Uygur Autonomous Region, Luntai County | 28 April 2018 | Wenwen Li, Yanan Wang | CAG 38 | CAG | China (Xinjiang) |
| *P. armeniaca* L. cv. 'yahelikeyulvke' | China, Xinjiang Uygur Autonomous Region, Luntai County | 28 April 2018 | Wenwen Li, Yanan Wang | CAG 43 | CAG | China (Xinjiang) |
| *P. armeniaca* L. cv. 'wanshuxing' | China, Xinjiang Uygur Autonomous Region, Luntai County | 28 April 2018 | Wenwen Li, Yanan Wang | CAG 20 | CAG | China (Xinjiang) |
| *P. armeniaca* L. cv. 'kezidalazi' | China, Xinjiang Uygur Autonomous Region, Luntai County | 28 April 2018 | Wenwen Li, Yanan Wang | CAG 33 | CAG | China (Xinjiang) |
| *P. armeniaca* L. cv. 'lajiaoxing' | China, Xinjiang Uygur Autonomous Region, Luntai County | 28 April 2018 | Wenwen Li, Yanan Wang | CAG 37 | CAG | China (Xinjiang) |
| *P. armeniaca* L. cv. 'hongdaike' | China, Xinjiang Uygur Autonomous Region, Luntai County | 28 April 2018 | Wenwen Li, Yanan Wang | CAG 10 | CAG | China (Xinjiang) |
| *P. armeniaca* L. cv. 'saimaiti' | China, Xinjiang Uygur Autonomous Region, Luntai County | 28 April 2018 | Wenwen Li, Yanan Wang | CAG 19 | CAG | China (Xinjiang) |
| *P. armeniaca* L. cv. 'huangqiligan' | China, Xinjiang Uygur Autonomous Region, Luntai County | 28 April 2018 | Wenwen Li, Yanan Wang | CAG 35 | CAG | China (Xinjiang) |
| *P. armeniaca* L. cv. 'kabakehuanna' | China, Xinjiang Uygur Autonomous Region, Luntai County | 28 April 2018 | Wenwen Li, Yanan Wang | CAG 03 | CAG | China (Xinjiang) |
| *P. armeniaca* L. cv. 'akedalazi' | China, Xinjiang Uygur Autonomous Region, Luntai County | 28 April 2018 | Wenwen Li, Yanan Wang | CAG 32 | CAG | China (Xinjiang) |
| *P. armeniaca* L. cv. 'xiaowuyuexing' | China, Xinjiang Uygur Autonomous Region, Luntai County | 28 April 2018 | Wenwen Li, Yanan Wang | CAG 47 | CAG | China (Xinjiang) |
| *P. armeniaca* L. cv. 'mantouyulvke' | China, Xinjiang Uygur Autonomous Region, Luntai County | 28 April 2018 | Wenwen Li, Yanan Wang | CAG 17 | CAG | China (Xinjiang) |
| *P. armeniaca* L. cv. 'suluke' | China, Xinjiang Uygur Autonomous Region, Luntai County | 28 April 2018 | Wenwen Li, Yanan Wang | CAG 34 | CAG | China (Xinjiang) |
| *P. armeniaca* L. cv. 'kabakeximixi' | China, Xinjiang Uygur Autonomous Region, Luntai County | 28 April 2018 | Wenwen Li, Yanan Wang | CAG 36 | CAG | China (Xinjiang) |
| *P. armeniaca* L. cv. 'yiliakeyulvke' | China, Xinjiang Uygur Autonomous Region, Luntai County | 28 April 2018 | Wenwen Li, Yanan Wang | CAG 45 | CAG | China (Xinjiang) |
| *P. armeniaca* L. cv. 'honghebaoxing' | China, Xinjiang Uygur Autonomous Region, Luntai County | 28 April 2018 | Wenwen Li, Yannan Wang | NCG 01 | NCG | China (Shandong) |
| *P. armeniaca* L. cv. 'erzhuanzi' | China, Xinjiang Uygur Autonomous Region, Luntai County | 28 April 2018 | Wenwen Li, Yannan Wang | NCG 02 | NCG | China (Shanxi) |
| *P. armeniaca* L. cv. 'zhupishuixing' | China, Xinjiang Uygur Autonomous Region, Luntai County | 28 April 2018 | Wenwen Li, Yannan Wang | NCG 03 | NCG | China (Gansu) |
| *P. armeniaca* L. cv. 'hongyuxing' | China, Xinjiang Uygur Autonomous Region, Luntai County | 28 April 2018 | Wenwen Li, Yannan Wang | NCG 04 | NCG | China (Shandong) |
| *P. armeniaca* L. cv. 'yinxiangbai' | China, Xinjiang Uygur Autonomous Region, Luntai County | 28 April 2018 | Wenwen Li, Yannan Wang | NCG 05 | NCG | China (Shanxi) |
| *P. armeniaca* L. cv. 'yeyinbai' | China, Xinjiang Uygur Autonomous Region, Luntai County | 28 April 2018 | Wenwen Li, Yannan Wang | NCG 06 | NCG | China (Liaoning) |
| *P. armeniaca* L. cv. 'zhanggongyuandajiexing' | China, Xinjiang Uygur Autonomous Region, Luntai County | 28 April 2018 | Wenwen Li, Yannan Wang | NCG 07 | NCG | China (Shanxi) |
| *P. armeniaca* L. cv. 'erhuacaoxing' | China, Xinjiang Uygur Autonomous Region, Luntai County | 28 April 2018 | Wenwen Li, Yannan Wang | NCG 08 | NCG | China (Shandong) |
| *P. armeniaca* L. cv. 'huangkouwai' | China, Xinjiang Uygur Autonomous Region, Luntai County | 28 April 2018 | Wenwen Li, Yannan Wang | NCG 09 | NCG | China (Ningxia) |
| *P. armeniaca* L. cv. 'manaoxing' | China, Xinjiang Uygur Autonomous Region, Luntai County | 28 April 2018 | Wenwen Li, Yannan Wang | NCG 10 | NCG | China (Shandong) |
| *P. armeniaca* L. cv. 'jinshahong 1' | China, Xinjiang Uygur Autonomous Region, Luntai County | 28 April 2018 | Wenwen Li, Yannan Wang | NCG 11 | NCG | China (Liaoning) |
| *P. armeniaca* L. cv. 'jiamaihuang' | China, Xinjiang Uygur Autonomous Region, Luntai County | 28 April 2018 | Wenwen Li, Yannan Wang | NCG 12 | NCG | China (Shandong) |
| *P. armeniaca* L. cv. 'chaoxianbaixing' | China, Xinjiang Uygur Autonomous Region, Luntai County | 28 April 2018 | Wenwen Li, Yannan Wang | NCG 13 | NCG | North Korea |
| *P. armeniaca* L. cv. 'early orange' | China, Liaoning Province, Xiongyue Town | 2-May-18 | Qiuping Zhang | EG 01 | EG | North America |
| *P. armeniaca* L. cv. 'harcot' | China, Liaoning Province, Xiongyue Town | 2-May-18 | Qiuping Zhang | EG 02 | EG | North America |
| *P. armeniaca* L. cv. 'jintaiyang' | China, Liaoning Province, Xiongyue Town | 2-May-18 | Qiuping Zhang | EG 03 | EG | North America |
| *P. armeniaca* L. cv. 'meiguo 2' | China, Liaoning Province, Xiongyue Town | 2-May-18 | Qiuping Zhang | EG 04 | EG | America |
| *P. armeniaca* L. cv. 'katy' | China, Liaoning Province, Xiongyue Town | 2-May-18 | Qiuping Zhang | EG 05 | EG | U.S.A (California) |
| *P. armeniaca* L. cv. 'pisala' | China, Liaoning Province, Xiongyue Town | 2-May-18 | Qiuping Zhang | EG 06 | EG | Italy |
| *P. armeniaca* L. cv. 'tyrinthos' | China, Liaoning Province, Xiongyue Town | 2-May-18 | Qiuping Zhang | EG 07 | EG | Italy |
| *P. armeniaca* L. cv. 'bergero' | China, Liaoning Province, Xiongyue Town | 2-May-18 | Qiuping Zhang | EG 08 | EG | France |
| Wild |  |  |  |  |  |  |
| *P. armeniaca* L. | China, Xinjiang Uygur Autonomous Region, Huocheng County | 8-May-18 | Kang Liao, Wenwen Li, Liqiang Liu, Yanan Wang | DZG hcmd 01 | DZG | China (Huocheng, Xinjiang) |
| *P. armeniaca* L. | China, Xinjiang Uygur Autonomous Region, Huocheng County | 8-May-18 | Kang Liao, Wenwen Li, Liqiang Liu, Yanan Wang | DZG hcmd 02 | DZG | China (Huocheng, Xinjiang) |
| *P. armeniaca* L. | China, Xinjiang Uygur Autonomous Region, Huocheng County | 8-May-18 | Kang Liao, Wenwen Li, Liqiang Liu, Yanan Wang | DZG hcmd 03 | DZG | China (Huocheng, Xinjiang) |
| *P. armeniaca* L. | China, Xinjiang Uygur Autonomous Region, Huocheng County | 8-May-18 | Kang Liao, Wenwen Li, Liqiang Liu, Yanan Wang | DZG hcmd 04 | DZG | China (Huocheng, Xinjiang) |
| *P. armeniaca* L. | China, Xinjiang Uygur Autonomous Region, Huocheng County | 8-May-18 | Kang Liao, Wenwen Li, Liqiang Liu, Yanan Wang | DZG hcmd 05 | DZG | China (Huocheng, Xinjiang) |
| *P. armeniaca* L. | China, Xinjiang Uygur Autonomous Region, Huocheng County | 8-May-18 | Kang Liao, Wenwen Li, Liqiang Liu, Yanan Wang | DZG hcmd 06 | DZG | China (Huocheng, Xinjiang) |
| *P. armeniaca* L. | China, Xinjiang Uygur Autonomous Region, Huocheng County | 8-May-18 | Kang Liao, Wenwen Li, Liqiang Liu, Yanan Wang | DZG hcmd 07 | DZG | China (Huocheng, Xinjiang) |
| *P. armeniaca* L. | China, Xinjiang Uygur Autonomous Region, Huocheng County | 8-May-18 | Kang Liao, Wenwen Li, Liqiang Liu, Yanan Wang | DZG hcmd 08 | DZG | China (Huocheng, Xinjiang) |
| *P. armeniaca* L. | China, Xinjiang Uygur Autonomous Region, Huocheng County | 8-May-18 | Kang Liao, Wenwen Li, Liqiang Liu, Yanan Wang | DZG hcmd 09 | DZG | China (Huocheng, Xinjiang) |
| *P. armeniaca* L. | China, Xinjiang Uygur Autonomous Region, Huocheng County | 8-May-18 | Kang Liao, Wenwen Li, Liqiang Liu, Yanan Wang | DZG hcmd 11 | DZG | China (Huocheng, Xinjiang) |
| *P. armeniaca* L. | China, Xinjiang Uygur Autonomous Region, Huocheng County | 8-May-18 | Kang Liao, Wenwen Li, Liqiang Liu, Yanan Wang | DZG hcmd 12 | DZG | China (Huocheng, Xinjiang) |
| *P. armeniaca* L. | China, Xinjiang Uygur Autonomous Region, Huocheng County | 8-May-18 | Kang Liao, Wenwen Li, Liqiang Liu, Yanan Wang | DZG hcmd 13 | DZG | China (Huocheng, Xinjiang) |
| *P. armeniaca* L. | China, Xinjiang Uygur Autonomous Region, Huocheng County | 8-May-18 | Kang Liao, Wenwen Li, Liqiang Liu, Yanan Wang | DZG hcmd 14 | DZG | China (Huocheng, Xinjiang) |
| *P. armeniaca* L. | China, Xinjiang Uygur Autonomous Region, Huocheng County | 8-May-18 | Kang Liao, Wenwen Li, Liqiang Liu, Yanan Wang | DZG hcmd 15 | DZG | China (Huocheng, Xinjiang) |
| *P. armeniaca* L. | China, Xinjiang Uygur Autonomous Region, Huocheng County | 8-May-18 | Kang Liao, Wenwen Li, Liqiang Liu, Yanan Wang | DZG hcmd 16 | DZG | China (Huocheng, Xinjiang) |
| *P. armeniaca* L. | China, Xinjiang Uygur Autonomous Region, Huocheng County | 8-May-18 | Kang Liao, Wenwen Li, Liqiang Liu, Yanan Wang | DZG hcy 17 | DZG | China (Huocheng, Xinjiang) |
| *P. armeniaca* L. | China, Xinjiang Uygur Autonomous Region, Huocheng County | 8-May-18 | Kang Liao, Wenwen Li, Liqiang Liu, Yanan Wang | DZG hcy 18 | DZG | China (Huocheng, Xinjiang) |
| *P. armeniaca* L. | China, Xinjiang Uygur Autonomous Region, Huocheng County | 8-May-18 | Kang Liao, Wenwen Li, Liqiang Liu, Yanan Wang | DZG hcy 19 | DZG | China (Huocheng, Xinjiang) |
| *P. armeniaca* L. | China, Xinjiang Uygur Autonomous Region, Huocheng County | 8-May-18 | Kang Liao, Wenwen Li, Liqiang Liu, Yanan Wang | DZG hcy 20 | DZG | China (Huocheng, Xinjiang) |
| *P. armeniaca* L. | China, Xinjiang Uygur Autonomous Region, Huocheng County | 8-May-18 | Kang Liao, Wenwen Li, Liqiang Liu, Yanan Wang | DZG hcy 21 | DZG | China (Huocheng, Xinjiang) |
| *P. armeniaca* L. | China, Xinjiang Uygur Autonomous Region, Huocheng County | 8-May-18 | Kang Liao, Wenwen Li, Liqiang Liu, Yanan Wang | DZG hcy 22 | DZG | China (Huocheng, Xinjiang) |
| *P. armeniaca* L. | China, Xinjiang Uygur Autonomous Region, Huocheng County | 8-May-18 | Kang Liao, Wenwen Li, Liqiang Liu, Yanan Wang | DZG hcy 23 | DZG | China (Huocheng, Xinjiang) |
| *P. armeniaca* L. | China, Xinjiang Uygur Autonomous Region, Huocheng County | 8-May-18 | Kang Liao, Wenwen Li, Liqiang Liu, Yanan Wang | DZG hcy 24 | DZG | China (Huocheng, Xinjiang) |
| *P. armeniaca* L. | China, Xinjiang Uygur Autonomous Region, Huocheng County | 8-May-18 | Kang Liao, Wenwen Li, Liqiang Liu, Yanan Wang | DZG hcy 25 | DZG | China (Huocheng, Xinjiang) |
| *P. armeniaca* L. | China, Xinjiang Uygur Autonomous Region, Huocheng County | 8-May-18 | Kang Liao, Wenwen Li, Liqiang Liu, Yanan Wang | DZG hcm 26 | DZG | China (Huocheng, Xinjiang) |
| *P. armeniaca* L. | China, Xinjiang Uygur Autonomous Region, Huocheng County | 8-May-18 | Kang Liao, Wenwen Li, Liqiang Liu, Yanan Wang | DZG hcm 27 | DZG | China (Huocheng, Xinjiang) |
| *P. armeniaca* L. | China, Xinjiang Uygur Autonomous Region, Huocheng County | 8-May-18 | Kang Liao, Wenwen Li, Liqiang Liu, Yanan Wang | DZG hcm 28 | DZG | China (Huocheng, Xinjiang) |
| *P. armeniaca* L. | China, Xinjiang Uygur Autonomous Region, Huocheng County | 8-May-18 | Kang Liao, Wenwen Li, Liqiang Liu, Yanan Wang | DZG hcm 29 | DZG | China (Huocheng, Xinjiang) |
| *P. armeniaca* L. | China, Xinjiang Uygur Autonomous Region, Huocheng County | 8-May-18 | Kang Liao, Wenwen Li, Liqiang Liu, Yanan Wang | DZG hcm 30 | DZG | China (Huocheng, Xinjiang) |
| *P. armeniaca* L. | China, Xinjiang Uygur Autonomous Region, Huocheng County | 8-May-18 | Kang Liao, Wenwen Li, Liqiang Liu, Yanan Wang | DZG hcm 31 | DZG | China (Huocheng, Xinjiang) |
| *P. armeniaca* L. | China, Xinjiang Uygur Autonomous Region, Huocheng County | 8-May-18 | Kang Liao, Wenwen Li, Liqiang Liu, Yanan Wang | DZG hcm 32 | DZG | China (Huocheng, Xinjiang) |
| *P. armeniaca* L. | China, Xinjiang Uygur Autonomous Region, Huocheng County | 8-May-18 | Kang Liao, Wenwen Li, Liqiang Liu, Yanan Wang | DZG hcm 33 | DZG | China (Huocheng, Xinjiang) |
| *P. armeniaca* L. | China, Xinjiang Uygur Autonomous Region, Huocheng County | 8-May-18 | Kang Liao, Wenwen Li, Liqiang Liu, Yanan Wang | DZG hcm 34 | DZG | China (Huocheng, Xinjiang) |
| *P. armeniaca* L. | China, Xinjiang Uygur Autonomous Region, Yining County | 7-May-18 | Kang Liao, Wenwen Li, Liqiang Liu, Yanan Wang | DZG yn 35 | DZG | China (Yining, Xinjiang) |
| *P. armeniaca* L. | China, Xinjiang Uygur Autonomous Region, Yining County | 7-May-18 | Kang Liao, Wenwen Li, Liqiang Liu, Yanan Wang | DZG yn 36 | DZG | China (Yining, Xinjiang) |
| *P. armeniaca* L. | China, Xinjiang Uygur Autonomous Region, Yining County | 7-May-18 | Kang Liao, Wenwen Li, Liqiang Liu, Yanan Wang | DZG yn 37 | DZG | China (Yining, Xinjiang) |
| *P. armeniaca* L. | China, Xinjiang Uygur Autonomous Region, Yining County | 7-May-18 | Kang Liao, Wenwen Li, Liqiang Liu, Yanan Wang | DZG yn 38 | DZG | China (Yining, Xinjiang) |
| *P. armeniaca* L. | China, Xinjiang Uygur Autonomous Region, Yining County | 7-May-18 | Kang Liao, Wenwen Li, Liqiang Liu, Yanan Wang | DZG yn 39 | DZG | China (Yining, Xinjiang) |
| *P. armeniaca* L. | China, Xinjiang Uygur Autonomous Region, Yining County | 7-May-18 | Kang Liao, Wenwen Li, Liqiang Liu, Yanan Wang | DZG yn 40 | DZG | China (Yining, Xinjiang) |
| *P. armeniaca* L. | China, Xinjiang Uygur Autonomous Region, Yining County | 7-May-18 | Kang Liao, Wenwen Li, Liqiang Liu, Yanan Wang | DZG yn 41 | DZG | China (Yining, Xinjiang) |
| *P. armeniaca* L. | China, Xinjiang Uygur Autonomous Region, Yining County | 7-May-18 | Kang Liao, Wenwen Li, Liqiang Liu, Yanan Wang | DZG yn 42 | DZG | China (Yining, Xinjiang) |
| *P. armeniaca* L. | China, Xinjiang Uygur Autonomous Region, Yining County | 7-May-18 | Kang Liao, Wenwen Li, Liqiang Liu, Yanan Wang | DZG yn 43 | DZG | China (Yining, Xinjiang) |
| *P. armeniaca* L. | China, Xinjiang Uygur Autonomous Region, Yining County | 7-May-18 | Kang Liao, Wenwen Li, Liqiang Liu, Yanan Wang | DZG yn 44 | DZG | China (Yining, Xinjiang) |
| *P. armeniaca* L. | China, Xinjiang Uygur Autonomous Region, Yining County | 7-May-18 | Kang Liao, Wenwen Li, Liqiang Liu, Yanan Wang | DZG yn 45 | DZG | China (Yining, Xinjiang) |
| *P. armeniaca* L. | China, Xinjiang Uygur Autonomous Region, Yining County | 7-May-18 | Kang Liao, Wenwen Li, Liqiang Liu, Yanan Wang | DZG yn 46 | DZG | China (Yining, Xinjiang) |
| *P. armeniaca* L. | China, Xinjiang Uygur Autonomous Region, Yining County | 7-May-18 | Kang Liao, Wenwen Li, Liqiang Liu, Yanan Wang | DZG yn 47 | DZG | China (Yining, Xinjiang) |
| *P. armeniaca* L. | China, Xinjiang Uygur Autonomous Region, Yining County | 7-May-18 | Kang Liao, Wenwen Li, Liqiang Liu, Yanan Wang | DZG yn 48 | DZG | China (Yining, Xinjiang) |
| *P. armeniaca* L. | China, Xinjiang Uygur Autonomous Region, Yining County | 7-May-18 | Kang Liao, Wenwen Li, Liqiang Liu, Yanan Wang | DZG yn 49 | DZG | China (Yining, Xinjiang) |
| *P. armeniaca* L. | China, Xinjiang Uygur Autonomous Region, Gongliu County | 5-May-18 | Kang Liao, Wenwen Li, Liqiang Liu, Yanan Wang | DZG glb50 | DZG | China (Gongliu, Xinjiang) |
| *P. armeniaca* L. | China, Xinjiang Uygur Autonomous Region, Gongliu County | 5-May-18 | Kang Liao, Wenwen Li, Liqiang Liu, Yanan Wang | DZG glb51 | DZG | China (Gongliu, Xinjiang) |
| *P. armeniaca* L. | China, Xinjiang Uygur Autonomous Region, Gongliu County | 5-May-18 | Kang Liao, Wenwen Li, Liqiang Liu, Yanan Wang | DZG glb52 | DZG | China (Gongliu, Xinjiang) |
| *P. armeniaca* L. | China, Xinjiang Uygur Autonomous Region, Gongliu County | 5-May-18 | Kang Liao, Wenwen Li, Liqiang Liu, Yanan Wang | DZG glb53 | DZG | China (Gongliu, Xinjiang) |
| *P. armeniaca* L. | China, Xinjiang Uygur Autonomous Region, Gongliu County | 5-May-18 | Kang Liao, Wenwen Li, Liqiang Liu, Yanan Wang | DZG glb54 | DZG | China (Gongliu, Xinjiang) |
| *P. armeniaca* L. | China, Xinjiang Uygur Autonomous Region, Gongliu County | 5-May-18 | Kang Liao, Wenwen Li, Liqiang Liu, Yanan Wang | DZG glb55 | DZG | China (Gongliu, Xinjiang) |
| *P. armeniaca* L. | China, Xinjiang Uygur Autonomous Region, Gongliu County | 5-May-18 | Kang Liao, Wenwen Li, Liqiang Liu, Yanan Wang | DZG glb56 | DZG | China (Gongliu, Xinjiang) |
| *P. armeniaca* L. | China, Xinjiang Uygur Autonomous Region, Gongliu County | 5-May-18 | Kang Liao, Wenwen Li, Liqiang Liu, Yanan Wang | DZG glb57 | DZG | China (Gongliu, Xinjiang) |
| *P. armeniaca* L. | China, Xinjiang Uygur Autonomous Region, Gongliu County | 5-May-18 | Kang Liao, Wenwen Li, Liqiang Liu, Yanan Wang | DZG glb58 | DZG | China (Gongliu, Xinjiang) |
| *P. armeniaca* L. | China, Xinjiang Uygur Autonomous Region, Gongliu County | 5-May-18 | Kang Liao, Wenwen Li, Liqiang Liu, Yanan Wang | DZG glb59 | DZG | China (Gongliu, Xinjiang) |
| *P. armeniaca* L. | China, Xinjiang Uygur Autonomous Region, Gongliu County | 5-May-18 | Kang Liao, Wenwen Li, Liqiang Liu, Yanan Wang | DZG gld 60 | DZG | China (Gongliu, Xinjiang) |
| *P. armeniaca* L. | China, Xinjiang Uygur Autonomous Region, Gongliu County | 5-May-18 | Kang Liao, Wenwen Li, Liqiang Liu, Yanan Wang | DZG gld 61 | DZG | China (Gongliu, Xinjiang) |
| *P. armeniaca* L. | China, Xinjiang Uygur Autonomous Region, Gongliu County | 5-May-18 | Kang Liao, Wenwen Li, Liqiang Liu, Yanan Wang | DZG gld 62 | DZG | China (Gongliu, Xinjiang) |
| *P. armeniaca* L. | China, Xinjiang Uygur Autonomous Region, Gongliu County | 5-May-18 | Kang Liao, Wenwen Li, Liqiang Liu, Yanan Wang | DZG gld 63 | DZG | China (Gongliu, Xinjiang) |
| *P. armeniaca* L. | China, Xinjiang Uygur Autonomous Region, Gongliu County | 5-May-18 | Kang Liao, Wenwen Li, Liqiang Liu, Yanan Wang | DZG gld 64 | DZG | China (Gongliu, Xinjiang) |
| *P. armeniaca* L. | China, Xinjiang Uygur Autonomous Region, Gongliu County | 5-May-18 | Kang Liao, Wenwen Li, Liqiang Liu, Yanan Wang | DZG gld 65 | DZG | China (Gongliu, Xinjiang) |
| *P. armeniaca* L. | China, Xinjiang Uygur Autonomous Region, Gongliu County | 5-May-18 | Kang Liao, Wenwen Li, Liqiang Liu, Yanan Wang | DZG gld 66 | DZG | China (Gongliu, Xinjiang) |
| *P. armeniaca* L. | China, Xinjiang Uygur Autonomous Region, Xinyuan County | 4-May-18 | Kang Liao, Wenwen Li, Liqiang Liu, Yanan Wang | DZG xyt67 | DZG | China (Xinyuan, Xinjiang) |
| *P. armeniaca* L. | China, Xinjiang Uygur Autonomous Region, Xinyuan County | 4-May-18 | Kang Liao, Wenwen Li, Liqiang Liu, Yanan Wang | DZG xyt68 | DZG | China (Xinyuan, Xinjiang) |
| *P. armeniaca* L. | China, Xinjiang Uygur Autonomous Region, Xinyuan County | 4-May-18 | Kang Liao, Wenwen Li, Liqiang Liu, Yanan Wang | DZG xyt69 | DZG | China (Xinyuan, Xinjiang) |
| *P. armeniaca* L. | China, Xinjiang Uygur Autonomous Region, Xinyuan County | 4-May-18 | Kang Liao, Wenwen Li, Liqiang Liu, Yanan Wang | DZG xyt70 | DZG | China (Xinyuan, Xinjiang) |
| *P. armeniaca* L. | China, Xinjiang Uygur Autonomous Region, Xinyuan County | 4-May-18 | Kang Liao, Wenwen Li, Liqiang Liu, Yanan Wang | DZG xyt71 | DZG | China (Xinyuan, Xinjiang) |
| *P. armeniaca* L. | China, Xinjiang Uygur Autonomous Region, Xinyuan County | 4-May-18 | Kang Liao, Wenwen Li, Liqiang Liu, Yanan Wang | DZG xyt72 | DZG | China (Xinyuan, Xinjiang) |
| *P. armeniaca* L. | China, Xinjiang Uygur Autonomous Region, Xinyuan County | 4-May-18 | Kang Liao, Wenwen Li, Liqiang Liu, Yanan Wang | DZG xyt73 | DZG | China (Xinyuan, Xinjiang) |
| *P. armeniaca* L. | China, Xinjiang Uygur Autonomous Region, Xinyuan County | 4-May-18 | Kang Liao, Wenwen Li, Liqiang Liu, Yanan Wang | DZG xyt74 | DZG | China (Xinyuan, Xinjiang) |
| *P. armeniaca* L. | China, Xinjiang Uygur Autonomous Region, Xinyuan County | 4-May-18 | Kang Liao, Wenwen Li, Liqiang Liu, Yanan Wang | DZG xyt75 | DZG | China (Xinyuan, Xinjiang) |
| *P. armeniaca* L. | China, Xinjiang Uygur Autonomous Region, Xinyuan County | 4-May-18 | Kang Liao, Wenwen Li, Liqiang Liu, Yanan Wang | DZG xyt76 | DZG | China (Xinyuan, Xinjiang) |
| *P. armeniaca* L. | China, Xinjiang Uygur Autonomous Region, Xinyuan County | 4-May-18 | Kang Liao, Wenwen Li, Liqiang Liu, Yanan Wang | DZG xyt77 | DZG | China (Xinyuan, Xinjiang) |
| *P. armeniaca* L. | China, Xinjiang Uygur Autonomous Region, Xinyuan County | 4-May-18 | Kang Liao, Wenwen Li, Liqiang Liu, Yanan Wang | DZG xya 78 | DZG | China (Xinyuan, Xinjiang) |
| *P. armeniaca* L. | China, Xinjiang Uygur Autonomous Region, Xinyuan County | 4-May-18 | Kang Liao, Wenwen Li, Liqiang Liu, Yanan Wang | DZG xya 79 | DZG | China (Xinyuan, Xinjiang) |
| *P. armeniaca* L. | China, Xinjiang Uygur Autonomous Region, Xinyuan County | 4-May-18 | Kang Liao, Wenwen Li, Liqiang Liu, Yanan Wang | DZG xya 80 | DZG | China (Xinyuan, Xinjiang) |
| *P. armeniaca* L. | China, Xinjiang Uygur Autonomous Region, Xinyuan County | 4-May-18 | Kang Liao, Wenwen Li, Liqiang Liu, Yanan Wang | DZG xyz 81 | DZG | China (Xinyuan, Xinjiang) |
| *P. armeniaca* L. | China, Xinjiang Uygur Autonomous Region, Xinyuan County | 4-May-18 | Kang Liao, Wenwen Li, Liqiang Liu, Yanan Wang | DZG xyz 82 | DZG | China (Xinyuan, Xinjiang) |
| *P. armeniaca* L. | China, Xinjiang Uygur Autonomous Region, Xinyuan County | 4-May-18 | Kang Liao, Wenwen Li, Liqiang Liu, Yanan Wang | DZG xyz 83 | DZG | China (Xinyuan, Xinjiang) |
| *P. armeniaca* L. | China, Xinjiang Uygur Autonomous Region, Xinyuan County | 4-May-18 | Kang Liao, Wenwen Li, Liqiang Liu, Yanan Wang | DZG xyz 84 | DZG | China (Xinyuan, Xinjiang) |
| *P. armeniaca* L. | China, Xinjiang Uygur Autonomous Region, Xinyuan County | 4-May-18 | Kang Liao, Wenwen Li, Liqiang Liu, Yanan Wang | DZG xyz 85 | DZG | China (Xinyuan, Xinjiang) |
| *P. armeniaca* L. | China, Xinjiang Uygur Autonomous Region, Xinyuan County | 4-May-18 | Kang Liao, Wenwen Li, Liqiang Liu, Yanan Wang | DZG xyz 86 | DZG | China (Xinyuan, Xinjiang) |
| *P. armeniaca* L. | China, Xinjiang Uygur Autonomous Region, Xinyuan County | 4-May-18 | Kang Liao, Wenwen Li, Liqiang Liu, Yanan Wang | DZG xyz 87 | DZG | China (Xinyuan, Xinjiang) |
| *P. armeniaca* L. | China, Xinjiang Uygur Autonomous Region, Xinyuan County | 4-May-18 | Kang Liao, Wenwen Li, Liqiang Liu, Yanan Wang | DZG xyz 88 | DZG | China (Xinyuan, Xinjiang) |
| *P. armeniaca* L. | China, Xinjiang Uygur Autonomous Region, Xinyuan County | 4-May-18 | Kang Liao, Wenwen Li, Liqiang Liu, Yanan Wang | DZG xyz 89 | DZG | China (Xinyuan, Xinjiang) |
| *P. armeniaca* L. | China, Xinjiang Uygur Autonomous Region, Xinyuan County | 4-May-18 | Kang Liao, Wenwen Li, Liqiang Liu, Yanan Wang | DZG xyz 90 | DZG | China (Xinyuan, Xinjiang) |
| *P. armeniaca* L. | China, Xinjiang Uygur Autonomous Region, Xinyuan County | 4-May-18 | Kang Liao, Wenwen Li, Liqiang Liu, Yanan Wang | DZG xyz 91 | DZG | China (Xinyuan, Xinjiang) |
| *P. sibirica* L. | China, Liaoning Province, Xiongyue Town | 2-May-18 | Qiuping Zhang | NAG 01 | NAG | China (Liaoning) |
| *P. sibirica* L. | China, Liaoning Province, Xiongyue Town | 2-May-18 | Qiuping Zhang | NAG 02 | NAG | China (Liaoning) |
| *P. sibirica* L. | China, Liaoning Province, Xiongyue Town | 2-May-18 | Qiuping Zhang | NAG 03 | NAG | China (Liaoning) |
| *P. sibirica* L. | China, Liaoning Province, Xiongyue Town | 2-May-18 | Qiuping Zhang | NAG 04 | NAG | China (Liaoning) |

**Table S2 Summary of the RAD sequencing in the 168 *Prunus* spp*.* accessions*.***

| Sample ID | Raw Base (Gbp) | Clean Base (Gbp) | Effective Rate (%) | Error Rate (%) | Q20 (%) | Q30 (%) | GC Content (%) | Mapping_rate (%) |
| --- | --- | --- | --- | --- | --- | --- | --- | --- |
| CAG 01 | 1.87 | 1.85 | 98.82 | 0.03 | 97.25 | 92.65 | 39.83 | 92.67 |
| CAG 02 | 2.24 | 2.19 | 98.03 | 0.03 | 96.66 | 91.86 | 38.77 | 92.71 |
| CAG 03 | 2.94 | 2.89 | 98.44 | 0.03 | 96.72 | 91.96 | 38.55 | 92.43 |
| CAG 04 | 1.93 | 1.91 | 98.85 | 0.03 | 97.45 | 93.14 | 39.56 | 92.42 |
| CAG 05 | 1.50 | 1.46 | 97.7 | 0.03 | 96.65 | 91.84 | 39.51 | 92.62 |
| CAG 06 | 2.09 | 2.06 | 98.76 | 0.03 | 95.68 | 89.61 | 39.57 | 88.70 |
| CAG 07 | 3.34 | 3.29 | 98.63 | 0.03 | 96.85 | 92.17 | 38.28 | 92.64 |
| CAG 08 | 1.76 | 1.67 | 94.84 | 0.03 | 97.41 | 93.12 | 39.38 | 91.46 |
| CAG 09 | 4.24 | 4.13 | 97.36 | 0.03 | 96.8 | 92.11 | 38.54 | 94.37 |
| CAG 10 | 1.38 | 1.37 | 98.88 | 0.03 | 97.27 | 92.75 | 39.68 | 92.55 |
| CAG 11 | 2.11 | 2.07 | 98.16 | 0.03 | 97.09 | 92.43 | 39.36 | 92.20 |
| CAG 12 | 1.61 | 1.58 | 98.13 | 0.03 | 97.03 | 92.27 | 39.64 | 92.19 |
| CAG 13 | 2.14 | 2.06 | 96.34 | 0.03 | 97.03 | 92.3 | 39.35 | 91.60 |
| CAG 14 | 8.61 | 8.52 | 99.03 | 0.03 | 96.69 | 91.46 | 41.02 | 92.35 |
| CAG 15 | 2.59 | 2.57 | 99.2 | 0.03 | 97.05 | 92.38 | 38.92 | 92.75 |
| CAG 16 | 2.17 | 2.14 | 98.8 | 0.03 | 96.75 | 91.59 | 39.07 | 91.52 |
| CAG 17 | 3.05 | 2.99 | 97.86 | 0.03 | 96.77 | 92.02 | 39.08 | 92.72 |
| CAG 18 | 2.04 | 1.97 | 96.25 | 0.03 | 97.36 | 92.95 | 38.94 | 91.97 |
| CAG 19 | 3.31 | 3.27 | 98.76 | 0.03 | 96.61 | 91.51 | 38.64 | 92.86 |
| CAG 20 | 1.17 | 1.16 | 99.01 | 0.03 | 97.3 | 92.79 | 39.48 | 92.75 |
| CAG 21 | 1.50 | 1.48 | 98.95 | 0.03 | 96.49 | 91.26 | 38.34 | 92.05 |
| CAG 22 | 1.91 | 1.89 | 98.72 | 0.03 | 96.97 | 92.27 | 40.61 | 92.81 |
| CAG 23 | 1.89 | 1.82 | 96.57 | 0.03 | 97.45 | 93.16 | 39.36 | 92.17 |
| CAG 24 | 1.75 | 1.73 | 99.13 | 0.03 | 97.52 | 93.28 | 39.61 | 93.03 |
| CAG 25 | 1.56 | 1.55 | 99.01 | 0.03 | 95.9 | 89.98 | 39.62 | 91.09 |
| CAG 26 | 1.23 | 1.22 | 98.98 | 0.03 | 95.69 | 89.66 | 39.63 | 91.14 |
| CAG 27 | 1.93 | 1.90 | 98.35 | 0.03 | 96.77 | 91.89 | 39.98 | 91.90 |
| CAG 28 | 1.75 | 1.73 | 98.7 | 0.03 | 97.26 | 92.64 | 39.32 | 92.52 |
| CAG 29 | 1.26 | 1.24 | 98.42 | 0.03 | 96.76 | 91.93 | 39.5 | 89.49 |
| CAG 30 | 1.69 | 1.67 | 98.88 | 0.03 | 96.78 | 91.93 | 39.31 | 91.22 |
| CAG 31 | 1.35 | 1.33 | 98.91 | 0.03 | 95.66 | 89.6 | 39.68 | 90.77 |
| CAG 32 | 2.12 | 2.09 | 98.59 | 0.03 | 96.97 | 92.28 | 40.08 | 92.60 |
| CAG 33 | 1.25 | 1.22 | 97.59 | 0.03 | 97.32 | 92.86 | 38.9 | 92.48 |
| CAG 34 | 1.67 | 1.62 | 96.76 | 0.03 | 97.43 | 93.11 | 39.09 | 92.36 |
| CAG 35 | 3.83 | 3.70 | 96.58 | 0.03 | 96.67 | 91.8 | 38.48 | 92.48 |
| CAG 36 | 1.66 | 1.64 | 99.04 | 0.03 | 97.19 | 92.52 | 39.89 | 92.44 |
| CAG 37 | 1.42 | 1.40 | 98.82 | 0.03 | 97.42 | 93.09 | 39.45 | 92.39 |
| CAG 38 | 1.36 | 1.33 | 97.34 | 0.03 | 97.38 | 93.02 | 39.05 | 92.07 |
| CAG 39 | 1.74 | 1.72 | 98.83 | 0.03 | 97.5 | 93.23 | 39.47 | 92.57 |
| CAG 40 | 2.73 | 2.70 | 98.84 | 0.03 | 96.61 | 91.55 | 38.17 | 92.56 |
| CAG 41 | 1.66 | 1.63 | 98.27 | 0.03 | 95.83 | 89.91 | 39.46 | 91.02 |
| CAG 42 | 10.00 | 9.88 | 98.85 | 0.03 | 96.84 | 91.72 | 40.28 | 91.92 |
| CAG 43 | 9.63 | 9.51 | 98.84 | 0.03 | 96.71 | 91.48 | 41.25 | 92.94 |
| CAG 44 | 2.52 | 2.50 | 98.95 | 0.03 | 96.56 | 91.44 | 38.36 | 92.48 |
| CAG 45 | 2.26 | 2.21 | 97.73 | 0.03 | 97.16 | 92.55 | 39.3 | 90.80 |
| CAG 46 | 2.72 | 2.66 | 97.75 | 0.03 | 97.15 | 92.53 | 39.14 | 91.86 |
| CAG 47 | 1.84 | 1.78 | 96.96 | 0.03 | 97.16 | 92.59 | 39.78 | 90.07 |
| CAG 48 | 1.78 | 1.73 | 97.24 | 0.03 | 95.8 | 89.39 | 38.99 | 90.61 |
| CAG 49 | 1.54 | 1.52 | 99.24 | 0.03 | 97.34 | 92.59 | 40.98 | 92.75 |
| CAG 50 | 1.77 | 1.74 | 97.94 | 0.03 | 95.71 | 89.25 | 40.05 | 91.80 |
| CAG 51 | 1.87 | 1.83 | 97.56 | 0.03 | 95.77 | 89.35 | 39.04 | 91.76 |
| CAG 52 | 1.99 | 1.96 | 98.67 | 0.03 | 97.32 | 92.83 | 39.36 | 92.79 |
| CAG 53 | 2.09 | 2.06 | 98.4 | 0.03 | 95.65 | 89.12 | 39.69 | 91.77 |
| DZG glb 50 | 2.91 | 2.84 | 97.68 | 0.03 | 96.88 | 92.12 | 38.3 | 91.58 |
| DZG glb 51 | 1.33 | 1.31 | 98.5 | 0.03 | 96.72 | 91.73 | 38.31 | 91.73 |
| DZG glb 52 | 1.28 | 1.26 | 98.38 | 0.03 | 96.72 | 91.8 | 38 | 91.92 |
| DZG glb 53 | 1.56 | 1.53 | 98.49 | 0.03 | 96.7 | 91.73 | 38.04 | 91.78 |
| DZG glb 54 | 1.87 | 1.85 | 99.08 | 0.03 | 96.75 | 91.82 | 37.88 | 91.65 |
| DZG glb 55 | 1.50 | 1.48 | 99 | 0.03 | 96.68 | 91.64 | 38.17 | 91.99 |
| DZG glb 56 | 2.97 | 2.92 | 98.53 | 0.03 | 96.74 | 91.78 | 37.62 | 92.17 |
| DZG glb 57 | 2.25 | 2.23 | 98.86 | 0.03 | 96.83 | 91.97 | 38.48 | 92.49 |
| DZG glb 58 | 3.38 | 3.34 | 98.89 | 0.03 | 96.81 | 91.95 | 38.13 | 92.47 |
| DZG glb 59 | 3.30 | 3.27 | 98.84 | 0.03 | 96.8 | 91.95 | 37.66 | 92.10 |
| DZG gld 60 | 1.75 | 1.71 | 98.07 | 0.03 | 96.68 | 91.54 | 37.67 | 91.90 |
| DZG gld 61 | 1.34 | 1.32 | 98.27 | 0.03 | 96.57 | 91.31 | 38.2 | 91.69 |
| DZG gld 62 | 2.68 | 2.64 | 98.55 | 0.03 | 96.69 | 91.53 | 38.03 | 91.74 |
| DZG gld 63 | 2.49 | 2.44 | 98.14 | 0.03 | 96.69 | 91.53 | 37.64 | 91.52 |
| DZG gld 64 | 2.59 | 2.53 | 97.74 | 0.03 | 96.26 | 90.58 | 38.11 | 91.59 |
| DZG gld 65 | 3.06 | 3.00 | 97.73 | 0.03 | 96.83 | 92.04 | 38.17 | 91.62 |
| DZG gld 66 | 2.52 | 2.47 | 98.22 | 0.03 | 96.81 | 91.9 | 38.31 | 91.98 |
| DZG hcm 26 | 2.00 | 1.98 | 99.17 | 0.03 | 96.81 | 91.97 | 38.05 | 92.19 |
| DZG hcm 27 | 2.19 | 2.16 | 98.85 | 0.03 | 96.76 | 91.91 | 38.11 | 92.21 |
| DZG hcm 28 | 2.24 | 2.21 | 98.59 | 0.03 | 96.5 | 91.28 | 38.27 | 92.10 |
| DZG hcm 29 | 3.62 | 3.55 | 97.87 | 0.03 | 96.34 | 91.07 | 38.05 | 92.01 |
| DZG hcm 30 | 3.18 | 3.12 | 98.19 | 0.03 | 96.3 | 90.89 | 37.77 | 91.88 |
| DZG hcm 31 | 3.68 | 3.60 | 97.95 | 0.03 | 96.36 | 91.08 | 37.73 | 91.86 |
| DZG hcm 32 | 1.59 | 1.57 | 98.63 | 0.03 | 96.2 | 90.71 | 37.67 | 91.86 |
| DZG hcm 33 | 1.81 | 1.78 | 98.37 | 0.03 | 96.22 | 90.83 | 37.84 | 91.70 |
| DZG hcm 34 | 1.80 | 1.77 | 98.34 | 0.03 | 96.22 | 90.78 | 37.59 | 91.79 |
| DZG hcmd 01 | 2.97 | 2.92 | 98.35 | 0.03 | 96.72 | 91.67 | 38.11 | 91.96 |
| DZG hcmd 02 | 3.35 | 3.28 | 97.87 | 0.03 | 96.93 | 92.16 | 37.58 | 91.99 |
| DZG hcmd 03 | 1.47 | 1.44 | 97.96 | 0.03 | 96.92 | 92.14 | 37.77 | 92.10 |
| DZG hcmd 04 | 3.38 | 3.32 | 98.3 | 0.03 | 96.38 | 91.06 | 37.89 | 91.95 |
| DZG hcmd 05 | 2.32 | 2.28 | 98.26 | 0.03 | 96.41 | 91.15 | 37.83 | 91.86 |
| DZG hcmd 06 | 1.64 | 1.62 | 98.35 | 0.03 | 96.31 | 90.94 | 38.16 | 92.03 |
| DZG hcmd 07 | 2.98 | 2.94 | 98.67 | 0.03 | 96.44 | 91.17 | 38.27 | 92.12 |
| DZG hcmd 08 | 3.29 | 3.23 | 98.26 | 0.03 | 96.42 | 91.14 | 38.12 | 91.99 |
| DZG hcmd 09 | 2.95 | 2.89 | 97.98 | 0.03 | 96.08 | 90.36 | 38.58 | 91.86 |
| DZG hcmd 11 | 1.71 | 1.69 | 98.78 | 0.03 | 97.46 | 93.12 | 39.41 | 91.59 |
| DZG hcmd 12 | 1.50 | 1.48 | 98.7 | 0.03 | 97.33 | 92.89 | 39.61 | 91.71 |
| DZG hcmd 13 | 1.81 | 1.77 | 97.92 | 0.03 | 97.38 | 93.02 | 39.17 | 91.49 |
| DZG hcmd 14 | 2.02 | 2.00 | 98.9 | 0.03 | 97.29 | 92.79 | 39.15 | 91.75 |
| DZG hcmd 15 | 1.19 | 1.18 | 99.15 | 0.03 | 97.38 | 93.02 | 39.24 | 92.10 |
| DZG hcmd 16 | 1.43 | 1.41 | 98.69 | 0.03 | 97.36 | 92.98 | 39.76 | 91.88 |
| DZG hcy 17 | 2.52 | 2.49 | 98.62 | 0.03 | 96.38 | 91.03 | 37.74 | 91.99 |
| DZG hcy 18 | 1.68 | 1.66 | 98.53 | 0.03 | 96.3 | 90.81 | 37.86 | 92.03 |
| DZG hcy 19 | 3.79 | 3.72 | 98.18 | 0.03 | 96.38 | 90.97 | 37.66 | 91.83 |
| DZG hcy 20 | 2.37 | 2.33 | 98.27 | 0.03 | 96.45 | 91.16 | 37.5 | 91.85 |
| DZG hcy 21 | 3.33 | 3.27 | 98.36 | 0.03 | 96.46 | 91.16 | 38.03 | 92.00 |
| DZG hcy 22 | 2.65 | 2.60 | 98.32 | 0.03 | 96.45 | 91.17 | 38.48 | 92.25 |
| DZG hcy 23 | 2.24 | 2.20 | 98.16 | 0.03 | 96.94 | 92.15 | 38.39 | 92.13 |
| DZG hcy 24 | 3.68 | 3.58 | 97.19 | 0.03 | 96.8 | 91.88 | 37.75 | 91.80 |
| DZG hcy 25 | 3.03 | 2.96 | 97.61 | 0.03 | 96.89 | 92.08 | 37.55 | 91.92 |
| DZG xya 78 | 1.96 | 1.95 | 99.09 | 0.03 | 96.49 | 91.5 | 38.03 | 91.96 |
| DZG xya 79 | 1.45 | 1.43 | 98.97 | 0.03 | 95.89 | 89.73 | 38.98 | 91.70 |
| DZG xya 80 | 1.56 | 1.54 | 98.99 | 0.03 | 95.73 | 89.4 | 37.62 | 91.60 |
| DZG xyt 67 | 2.90 | 2.86 | 98.88 | 0.03 | 97.15 | 92.8 | 38.12 | 92.32 |
| DZG xyt 68 | 2.15 | 2.13 | 98.9 | 0.03 | 97.2 | 92.91 | 38.07 | 91.97 |
| DZG xyt 69 | 1.50 | 1.48 | 98.96 | 0.03 | 97.12 | 92.75 | 37.77 | 92.03 |
| DZG xyt 70 | 2.11 | 2.09 | 99.12 | 0.03 | 97.22 | 92.91 | 38 | 92.13 |
| DZG xyt 71 | 3.22 | 3.18 | 98.82 | 0.03 | 97.19 | 92.91 | 38.02 | 92.11 |
| DZG xyt 72 | 2.65 | 2.61 | 98.6 | 0.03 | 96.92 | 92.24 | 37.92 | 92.36 |
| DZG xyt 73 | 3.33 | 3.28 | 98.44 | 0.03 | 96.61 | 91.75 | 38.33 | 91.88 |
| DZG xyt 74 | 2.58 | 2.55 | 98.84 | 0.03 | 96.59 | 91.63 | 37.7 | 92.36 |
| DZG xyt 75 | 3.75 | 3.70 | 98.65 | 0.03 | 96.68 | 91.81 | 37.98 | 92.27 |
| DZG xyt 76 | 1.57 | 1.55 | 99.1 | 0.03 | 96.48 | 91.45 | 37.84 | 91.93 |
| DZG xyt 77 | 1.36 | 1.34 | 98.92 | 0.03 | 96.5 | 91.53 | 37.65 | 91.60 |
| DZG xyz 81 | 4.27 | 4.22 | 98.75 | 0.03 | 95.75 | 89.47 | 37.73 | 91.77 |
| DZG xyz 82 | 2.12 | 2.10 | 98.95 | 0.03 | 95.88 | 89.72 | 37.52 | 91.63 |
| DZG xyz 83 | 3.56 | 3.52 | 98.92 | 0.03 | 95.89 | 89.72 | 37.72 | 91.74 |
| DZG xyz 84 | 3.17 | 3.13 | 98.89 | 0.03 | 95.85 | 89.69 | 37.5 | 91.70 |
| DZG xyz 85 | 2.35 | 2.32 | 98.69 | 0.03 | 96.75 | 91.79 | 37.91 | 92.06 |
| DZG xyz 86 | 3.44 | 3.37 | 97.87 | 0.03 | 96.66 | 91.6 | 38.08 | 92.22 |
| DZG xyz 87 | 2.60 | 2.55 | 98.07 | 0.03 | 96.75 | 91.79 | 37.74 | 92.02 |
| DZG xyz 88 | 2.19 | 2.17 | 98.73 | 0.03 | 96.58 | 91.42 | 37.52 | 92.15 |
| DZG xyz 89 | 3.24 | 3.18 | 98.27 | 0.03 | 96.78 | 91.86 | 37.74 | 92.07 |
| DZG xyz 90 | 1.19 | 1.17 | 98.3 | 0.03 | 96.72 | 91.75 | 37.9 | 91.98 |
| DZG xyz 91 | 1.83 | 1.79 | 97.79 | 0.03 | 96.62 | 91.41 | 37.73 | 91.46 |
| DZG yn 35 | 2.84 | 2.81 | 98.98 | 0.03 | 96.49 | 91.28 | 38.34 | 92.32 |
| DZG yn 36 | 4.25 | 4.18 | 98.37 | 0.03 | 96.34 | 91.03 | 38.06 | 92.06 |
| DZG yn 37 | 2.85 | 2.80 | 98.46 | 0.03 | 96.38 | 91.14 | 37.33 | 92.08 |
| DZG yn 38 | 3.05 | 3.02 | 99.12 | 0.03 | 96.31 | 90.91 | 38.11 | 92.22 |
| DZG yn 39 | 3.55 | 3.51 | 98.71 | 0.03 | 96.44 | 91.24 | 38.2 | 92.15 |
| DZG yn 40 | 1.50 | 1.48 | 98.76 | 0.03 | 96.4 | 91.16 | 37.56 | 92.33 |
| DZG yn 41 | 2.16 | 2.14 | 98.79 | 0.03 | 96.73 | 91.84 | 38.08 | 92.30 |
| DZG yn 42 | 1.19 | 1.18 | 98.73 | 0.03 | 96.77 | 91.93 | 38.45 | 91.99 |
| DZG yn 43 | 1.12 | 1.11 | 98.9 | 0.03 | 96.7 | 91.78 | 37.97 | 92.26 |
| DZG yn 44 | 2.27 | 2.24 | 98.9 | 0.03 | 97.22 | 92.9 | 38.02 | 91.94 |
| DZG yn 45 | 4.53 | 4.46 | 98.48 | 0.03 | 97.09 | 92.66 | 38.04 | 92.14 |
| DZG yn 46 | 3.57 | 3.52 | 98.66 | 0.03 | 97.15 | 92.79 | 38.04 | 92.04 |
| DZG yn 47 | 2.91 | 2.88 | 99.01 | 0.03 | 97.06 | 92.56 | 38.24 | 92.27 |
| DZG yn 48 | 3.78 | 3.74 | 98.84 | 0.03 | 97.16 | 92.84 | 37.8 | 92.16 |
| DZG yn 49 | 1.62 | 1.60 | 98.83 | 0.03 | 97.16 | 92.83 | 38.06 | 92.50 |
| NCG 01 | 3.33 | 3.27 | 98.22 | 0.03 | 96.49 | 91.32 | 38.94 | 92.21 |
| NCG 02 | 2.02 | 1.97 | 97.49 | 0.03 | 95.78 | 89.79 | 39.23 | 90.50 |
| NCG 03 | 2.05 | 2.01 | 98.01 | 0.03 | 96.91 | 92.19 | 39.73 | 91.31 |
| NCG 04 | 2.58 | 2.55 | 98.66 | 0.03 | 96.78 | 91.75 | 39.04 | 92.05 |
| NCG 05 | 1.68 | 1.66 | 98.93 | 0.03 | 96.98 | 92.31 | 39.15 | 92.03 |
| NCG 06 | 1.69 | 1.67 | 98.79 | 0.03 | 97.26 | 92.82 | 39.56 | 91.14 |
| NCG 07 | 2.24 | 2.20 | 98.13 | 0.03 | 96.67 | 91.9 | 38.3 | 91.94 |
| NCG 08 | 1.56 | 1.54 | 98.73 | 0.03 | 96.92 | 92.17 | 39.21 | 91.85 |
| NCG 09 | 1.87 | 1.83 | 97.65 | 0.03 | 96.75 | 91.94 | 38.47 | 91.96 |
| NCG 10 | 1.88 | 1.86 | 98.81 | 0.03 | 97.32 | 92.81 | 39.64 | 91.74 |
| NCG 11 | 1.61 | 1.60 | 99.27 | 0.03 | 97.34 | 92.62 | 40.09 | 90.94 |
| NCG 12 | 1.93 | 1.92 | 99.27 | 0.03 | 97.28 | 92.47 | 41.17 | 92.11 |
| NCG 13 | 1.77 | 1.75 | 98.95 | 0.03 | 97.22 | 92.69 | 39.36 | 91.53 |
| EG 01 | 2.01 | 1.99 | 98.93 | 0.03 | 97.09 | 92.44 | 39.73 | 91.71 |
| EG 02 | 1.64 | 1.62 | 99.08 | 0.03 | 97.14 | 92.54 | 39.22 | 91.82 |
| EG 03 | 1.35 | 1.33 | 98.47 | 0.03 | 96.84 | 91.88 | 38.53 | 91.60 |
| EG 04 | 2.06 | 2.00 | 97.19 | 0.03 | 96.88 | 92.06 | 40.29 | 91.54 |
| EG 05 | 2.23 | 2.20 | 98.81 | 0.03 | 97.16 | 92.58 | 39.27 | 91.51 |
| EG 06 | 1.40 | 1.38 | 98.5 | 0.03 | 95.81 | 89.44 | 39.25 | 91.62 |
| EG 07 | 1.24 | 1.22 | 98.3 | 0.03 | 95.73 | 89.3 | 39.01 | 92.18 |
| EG 08 | 1.61 | 1.60 | 98.9 | 0.03 | 97.1 | 92.5 | 37.97 | 91.71 |
| NAG 01 | 1.91 | 1.89 | 98.91 | 0.03 | 97.28 | 92.83 | 39.59 | 90.81 |
| NAG 02 | 1.41 | 1.40 | 99.15 | 0.03 | 97.12 | 92.5 | 40.05 | 91.49 |
| NAG 03 | 1.44 | 1.42 | 99.08 | 0.03 | 97.14 | 92.57 | 39.39 | 91.16 |
| NAG 04 | 1.46 | 1.44 | 99.21 | 0.03 | 97.15 | 92.56 | 38.82 | 91.16 |

**Table S3 The SNP mutation type for the five ecological groups****.** Ts/tv: Transition vs. transversion rate

| Statistics | NAG | CAG | DZG | EG | NCG |
| --- | --- | --- | --- | --- | --- |
| No. of transitions | 122104 | 250942 | 258546 | 171267 | 198097 |
| No. of transversions | 67872 | 139950 | 144157 | 95502 | 110833 |
| Ts/tv | 1.799 | 1.793 | 1.794 | 1.793 | 1.787 |
| Transitions rate | 0.556 | 0.558 | 0.558 | 0.558 | 0.559 |

**Table S4 List of genes in selective sweep regions involved in important traits of DZG and CAG accessions during domestication from wild to cultivated apricots.**

| Gene | Chromosome | Start position | End position | Length | Pathway | DZG | | CAG | | *F*st |
| --- | --- | --- | --- | --- | --- | --- | --- | --- | --- | --- |
|  |  |  |  |  |  | π | Tajima’s D | π value | Tajima’s D |  |
| XM_008235983.2 | chr5 | 23230004 | 23233213 | 3209 | ABA/carotenoid biosynthesis | 1.11E-05 | -1.079 | 1.34E-04 | 1.690 | 0.377 |
| XM_008240936.2 | chr7 | 10699786 | 10703406 | 3620 | Cellulose synthase | 1.89E-06 | -1.021 | 4.73E-05 | 1.777 | 0.304 |
| XM_008228061.2 | chr3 | 9754341 | 9758714 | 4373 | Serine and threonine metabolism | 9.07E-06 | -0.595 | 6.17E-05 | 1.083 | 0.256 |
| XM_008241536.2 | chr7 | 13901549 | 13905722 | 4173 | Serine and threonine metabolism | 7.33E-06 | -0.698 | 4.98E-05 | 1.926 | 0.433 |
| XM_008236207.1 | chr5 | 24348158 | 24352099 | 3941 | GTPase regulator activity | 1.73E-05 | -0.110 | 5.00E-05 | 1.937 | 0.259 |
| XM_008242126.2 | chr7 | 16667912 | 16673545 | 5633 | GTPase regulator activity | 7.55E-06 | -1.769 | 2.66E-04 | 3.201 | 0.295 |

**Table S5 List of genes in selective sweeps in DZG and CAG accessions during domestication from wild to cultivated apricots.**

| Gene_name | Chromosome | Start position | End position |
| --- | --- | --- | --- |
| XM_008242525.1 | chr1 | 20889248 | 20891389 |
| XM_008242537.1 | chr1 | 20892169 | 20897041 |
| XM_016797347.1 | chr1 | 20892220 | 20897041 |
| XM_008221931.1 | chr1 | 410785 | 416150 |
| XM_008223742.2 | chr2 | 19381210 | 19382862 |
| XM_008223743.1 | chr2 | 19386395 | 19388184 |
| XM_008223971.1 | chr2 | 20960033 | 20963529 |
| XM_008223972.1 | chr2 | 20960033 | 20963529 |
| XM_008223973.1 | chr2 | 20966215 | 20972559 |
| XM_008221127.1 | chr2 | 5283321 | 5293007 |
| XM_008221128.2 | chr2 | 5294192 | 5305986 |
| XM_008221608.1 | chr2 | 8215038 | 8238318 |
| XM_016792181.1 | chr2 | 8215644 | 8238318 |
| XM_008228176.2 | chr3 | 10315848 | 10318756 |
| XM_008228276.1 | chr3 | 10939338 | 10943489 |
| XM_008228277.1 | chr3 | 10939338 | 10943489 |
| XM_008228278.1 | chr3 | 10939338 | 10943489 |
| XM_008228369.2 | chr3 | 11571542 | 11573027 |
| XM_008228370.1 | chr3 | 11578843 | 11590119 |
| XM_008228488.1 | chr3 | 12474269 | 12484982 |
| XM_008228489.2 | chr3 | 12474269 | 12484259 |
| XM_008226899.2 | chr3 | 2395445 | 2397443 |
| XM_008227536.2 | chr3 | 5488873 | 5490620 |
| XM_008228059.2 | chr3 | 9744880 | 9751941 |
| XM_008228060.2 | chr3 | 9744880 | 9751941 |
| XM_008228061.2 | chr3 | 9754341 | 9758714 |
| XM_008231481.1 | chr4 | 15208318 | 15209679 |
| XM_008235983.2 | chr5 | 23230004 | 23233213 |
| XM_008236174.2 | chr5 | 24136329 | 24151859 |
| XM_008236175.1 | chr5 | 24155444 | 24159445 |
| XM_008236207.1 | chr5 | 24348158 | 24352099 |
| XM_016794323.1 | chr5 | 24352221 | 24353299 |
| XM_008236208.1 | chr5 | 24355740 | 24359280 |
| XM_008236209.2 | chr5 | 24356360 | 24359280 |
| XM_008237211.2 | chr6 | 2304832 | 2306619 |
| XM_008237212.1 | chr6 | 2308916 | 2312933 |
| XM_008237213.2 | chr6 | 2308953 | 2312933 |
| XM_008240924.2 | chr7 | 10644104 | 10650030 |
| XM_016795958.1 | chr7 | 10651601 | 10655203 |
| XM_008240925.2 | chr7 | 10651602 | 10654993 |
| XM_008240926.2 | chr7 | 10656122 | 10658721 |
| XM_008240936.2 | chr7 | 10699786 | 10703406 |
| XM_008240979.2 | chr7 | 10985498 | 10987283 |
| XM_008241052.1 | chr7 | 11516090 | 11517973 |
| XM_008241064.2 | chr7 | 11617939 | 11622191 |
| XM_008241067.2 | chr7 | 11624772 | 11629842 |
| XM_008241065.2 | chr7 | 11624772 | 11629789 |
| XM_008241536.2 | chr7 | 13901549 | 13905722 |
| XM_008242116.2 | chr7 | 16627994 | 16633405 |
| XM_008242117.1 | chr7 | 16633653 | 16637578 |
| XM_008242119.2 | chr7 | 16638011 | 16643424 |
| XM_008242126.2 | chr7 | 16667912 | 16673545 |
| XM_008242127.1 | chr7 | 16679474 | 16681557 |

**Table S6 List of genes in selective sweep regions involved in important traits of DZG and EG accessions during domestication from wild to cultivated apricots.**

| Gene | Chromosome | Start position | End position | Length | Pathway | DZG | | CAG | | *F*st |
| --- | --- | --- | --- | --- | --- | --- | --- | --- | --- | --- |
|  |  |  |  |  |  | π | Tajima’s D | π | Tajima’s D |  |
| XM_016791935.1 | chr2 | 40739440 | 40752176 | 12736 | Ethylene and ABA | 2.64E-05 | 0.538975 | 4.58E-05 | 1.03439 | 0.495502 |
| XM_008228774.2 | chr3 | 14502263 | 14503517 | 1254 | Ethylene and ABA | 2.87E-05 | 0.672733 | 0.000105 | 1.89943 | 0.535376 |
| XM_008234736.2 | chr5 | 16813323 | 16815208 | 1885 | Aroma development | 2.89E-05 | -0.233207 | 0.000163 | 1.11045 | 0.41648 |
| XM_008230648.1 | chr1 | 9271367 | 9273310 | 1943 | Aroma development | 4.37E-06 | -0.769856 | 5.33E-05 | 1.52862 | 0.73439 |
| XM_008235018.1 | chr5 | 18355840 | 18364390 | 8550 | Organic acid development | 1.06E-05 | -0.402853 | 5.25E-05 | 1.4737 | 0.508629 |
| XM_008238560.2 | chr1 | 18462519 | 18465910 | 3391 | Organic acid development | 1.63E-05 | -0.0623796 | 5.33E-05 | 1.52862 | 0.457116 |
| XM_008223642.2 | chr2 | 18666482 | 18668845 | 2363 | High-affinity transport system | 1.54E-05 | -0.117283 | 8.50E-05 | 1.05009 | 0.425747 |
| XM_008235020.1 | chr5 | 18366200 | 18367494 | 1294 | Stress tolerance | 1.06E-05 | -0.402853 | 5.25E-05 | 1.4737 | 0.508629 |
| XM_008224003.1 | chr2 | 21204702 | 21206541 | 1839 | Stress tolerance | 1.15E-05 | -0.344265 | 5.25E-05 | 1.4737 | 0.62601 |
| XM_008223640.1 | chr2 | 18658967 | 18661626 | 2659 | Pectin degradation | 1.54E-05 | -0.117283 | 8.50E-05 | 1.05009 | 0.425747 |
| XM_008227516.2 | chr3 | 6096670 | 6099586 | 2916 | Pathogen related | 3.30E-06 | -0.833603 | 5.00E-05 | 1.30896 | 0.648223 |
| XM_008231816.2 | chr4 | 17433345 | 17436494 | 3149 | Auxin related | 2.07E-05 | 0.201082 | 5.25E-05 | 1.4737 | 0.451148 |
| XM_008240728.1 | chr7 | 9384037 | 9386523 | 2486 | Auxin related | 2.11E-05 | -0.543294 | 0.000105 | 1.89943 | 0.64424 |
| XM_008229480.2 | chr3 | 24051230 | 24059038 | 7808 | Serine and threonine metabolism | 3.30E-06 | -0.833603 | 5.00E-05 | 1.30896 | 0.644246 |
| XM_016792850.1 | chr3 | 24051230 | 24058755 | 7525 | Serine and threonine metabolism | 3.30E-06 | -0.833603 | 5.00E-05 | 1.30896 | 0.644246 |
| XM_008223599.2 | chr2 | 18394839 | 18401064 | 6225 | Serine and threonine metabolism | 8.54E-06 | -0.522239 | 5.25E-05 | 1.4737 | 0.690406 |
| XM_008236070.2 | chr5 | 23610678 | 23614923 | 4245 | Serine and threonine metabolism | 2.56E-05 | 0.492916 | 4.58E-05 | 1.03439 | 0.507455 |
| XM_008236069.2 | chr5 | 23610678 | 23614923 | 4245 | Serine and threonine metabolism | 2.56E-05 | 0.492916 | 4.58E-05 | 1.03439 | 0.507455 |
| XM_016794701.1 | chr5 | 23610678 | 23614801 | 4123 | Serine and threonine metabolism | 2.56E-05 | 0.492916 | 4.58E-05 | 1.03439 | 0.507455 |
| XM_008236072.2 | chr5 | 23610678 | 23614027 | 3349 | Serine and threonine metabolism | 2.56E-05 | 0.492916 | 4.58E-05 | 1.03439 | 0.507455 |
| XM_008236071.2 | chr5 | 23610678 | 23614027 | 3349 | Serine and threonine metabolism | 2.56E-05 | 0.492916 | 4.58E-05 | 1.03439 | 0.507455 |
| XM_008238184.1 | chr6 | 7546811 | 7549234 | 2423 | Salicylic acid biosynthesis | 4.37E-06 | -0.769856 | 5.00E-05 | 1.30896 | 0.820439 |

**Table S8 List of genes in selective sweep regions involved in important traits of DZG and NCG accessions during domestication from wild to cultivated apricots.**

| c | Chromosome | Start position | End position | Length | Pathway | DZG | | CAG | | *F*st |
| --- | --- | --- | --- | --- | --- | --- | --- | --- | --- | --- |
|  |  |  |  |  |  | π | Tajima’s D | π | Tajima’s D |  |
| XM_008225204.1 | chr2 | 33026330 | 33030523 | 4193 | Sugar metabolism | 1.63E-05 | -6.24E-02 | 5.20E-05 | 1.61005 | 0.443927 |
| XM_008220307.2 | chr1 | 26172530 | 26182201 | 9671 | Sugar metabolism | 2.21E-06 | -0.898086 | 3.69E-05 | 0.668964 | 0.422107 |
| XM_008225204.1 | chr2 | 33026330 | 33030523 | 4193 | Sugar metabolism | 1.63E-05 | -6.24E-02 | 5.20E-05 | 1.61005 | 0.443927 |
| XM_008228014.2 | chr3 | 9346613 | 9349091 | 2478 | Sugar metabolism | 2.64E-05 | 0.538975 | 4.71E-05 | 1.30276 | 0.455596 |
| XM_008235018.1 | chr5 | 18355840 | 18364390 | 8550 | Organic acid development | 1.06E-05 | -0.402853 | 5.08E-05 | 1.53323 | 0.462455 |
| XM_008235020.1 | chr5 | 18366200 | 18367494 | 1294 | Stress tolerance | 1.06E-05 | -0.402853 | 5.08E-05 | 1.53323 | 0.462455 |
| XM_008240322.1 | chr1 | 19523148 | 19525588 | 2440 | Salicylic acid biosynthesis | 2.16E-05 | 0.251563 | 3.69E-05 | 0.668964 | 0.637553 |
| XM_016791935.1 | chr2 | 40739440 | 40752176 | 12736 | Ethylene and ABA | 2.64E-05 | 0.538975 | 4.92E-05 | 1.4372 | 0.413018 |
| XM_016792599.1 | chr2 | 40675101 | 40676926 | 1825 | Ethylene biosynthesis | 1.81E-05 | -0.663801 | 7.88E-05 | 1.07714 | 0.489872 |
| XM_016792168.1 | chr2 | 40678559 | 40682394 | 3835 | Ethylene biosynthesis | 1.81E-05 | -0.663801 | 7.88E-05 | 1.07714 | 0.489872 |
| XM_016792169.1 | chr2 | 40678559 | 40681939 | 3380 | Ethylene biosynthesis | 1.81E-05 | -0.663801 | 7.88E-05 | 1.07714 | 0.489872 |
| XM_016791935.1 | chr2 | 40739440 | 40752176 | 12736 | Ethylene biosynthesis | 2.64E-05 | 0.538975 | 4.92E-05 | 1.4372 | 0.413018 |
| XM_008222793.2 | chr2 | 14093093 | 14103018 | 9925 | GTPase regulator activity | 1.90E-05 | 0.097908 | 5.97E-05 | 0.297539 | 0.510407 |
| XM_016791409.1 | chr2 | 8287810 | 8292599 | 4789 | GTPase regulator activity | 1.80E-05 | -0.666782 | 6.68E-05 | 0.586745 | 0.441429 |
| XM_016791408.1 | chr2 | 8287810 | 8292599 | 4789 | GTPase regulator activity | 1.80E-05 | -0.666782 | 6.68E-05 | 0.586745 | 0.441429 |
| XM_008221686.2 | chr2 | 8287810 | 8292598 | 4788 | GTPase regulator activity | 1.80E-05 | -0.666782 | 6.68E-05 | 0.586745 | 0.441429 |
| XM_016791410.1 | chr2 | 8287810 | 8292492 | 4682 | GTPase regulator activity | 1.80E-05 | -0.666782 | 6.68E-05 | 0.586745 | 0.441429 |
| XM_008228510.1 | chr3 | 12717310 | 12720859 | 3549 | Serine and threonine metabolism | 1.80E-05 | -0.666782 | 6.68E-05 | 0.586745 | 0.441429 |
| XM_016793150.1 | chr3 | 12717387 | 12720859 | 3472 | Serine and threonine metabolism | 2.16E-05 | 0.251563 | 3.23E-05 | 0.380877 | 0.669521 |
| XM_016793151.1 | chr3 | 12722514 | 12726115 | 3601 | Serine and threonine metabolism | 2.16E-05 | 0.251563 | 3.23E-05 | 0.380877 | 0.669521 |
| XM_016793152.1 | chr3 | 12722514 | 12725706 | 3192 | Serine and threonine metabolism | 2.16E-05 | 0.251563 | 3.23E-05 | 0.380877 | 0.669521 |
| XM_008243793.2 | chr1 | 21707945 | 21711101 | 3156 | Serine and threonine metabolism | 9.55E-06 | -0.462178 | 5.17E-05 | 1.59084 | 0.531148 |
| XM_008246035.2 | chr1 | 22446796 | 22450136 | 3340 | Serine and threonine metabolism | 7.52E-06 | -0.583038 | 4.92E-05 | 1.4372 | 0.480362 |
| XM_016793469.1 | chr4 | 13054711 | 13059531 | 4820 | Serine and threonine metabolism | 6.48E-06 | -0.644574 | 5.08E-05 | 1.53323 | 0.555558 |
| XM_008236070.2 | chr5 | 23610678 | 23614923 | 4245 | Serine and threonine metabolism | 2.56E-05 | 0.492916 | 4.71E-05 | 1.30276 | 0.469039 |
| XM_008236069.2 | chr5 | 23610678 | 23614923 | 4245 | Serine and threonine metabolism | 2.56E-05 | 0.492916 | 4.71E-05 | 1.30276 | 0.469039 |
| XM_008236072.2 | chr5 | 23610678 | 23614027 | 3349 | Serine and threonine metabolism | 2.56E-05 | 0.492916 | 4.71E-05 | 1.30276 | 0.469039 |
| XM_008236071.2 | chr5 | 23610678 | 23614027 | 3349 | Serine and threonine metabolism | 2.56E-05 | 0.492916 | 4.71E-05 | 1.30276 | 0.469039 |
| XM_008236672.2 | chr5 | 25861559 | 25865015 | 3456 | Serine and threonine metabolism | 7.52E-06 | -0.583038 | 9.63E-05 | 1.79386 | 0.623863 |
| XM_008243126.2 | chr8 | 9001647 | 9003634 | 1987 | Purine metabolism | 5.43E-06 | -0.706847 | 4.71E-05 | 1.30276 | 0.485468 |
| XM_008240634.1 | chr7 | 8550082 | 8552160 | 2078 | Glucuronoxylan biosynthesis | 2.21E-06 | -0.898086 | 5.17E-05 | 1.59084 | 0.776136 |
| XM_008240728.1 | chr7 | 9384037 | 9386523 | 2486 | Auxin related | 2.11E-05 | -0.543294 | 9.42E-05 | 1.70585 | 0.700663 |
| XM_016794548.1 | chr5 | 25452160 | 25464833 | 12673 | Alanine, aspartate and glutamate metabolism | 1.19E-05 | -0.912268 | 4.92E-05 | 1.4372 | 0.679938 |
| XM_008227100.1 | chr3 | 3584816 | 3591037 | 6221 | Cellulose biosynthesis | 1.71E-05 | -0.704301 | 1.02E-04 | 2.00763 | 0.502837 |
| XM_008227516.2 | chr3 | 6096670 | 6099586 | 2916 | Pathogen related | 3.30E-06 | -0.833603 | 5.08E-05 | 5.08E-05 | 0.773435 |

**Fig. 1 The number of different groups divided corresponds to CV errors value.**


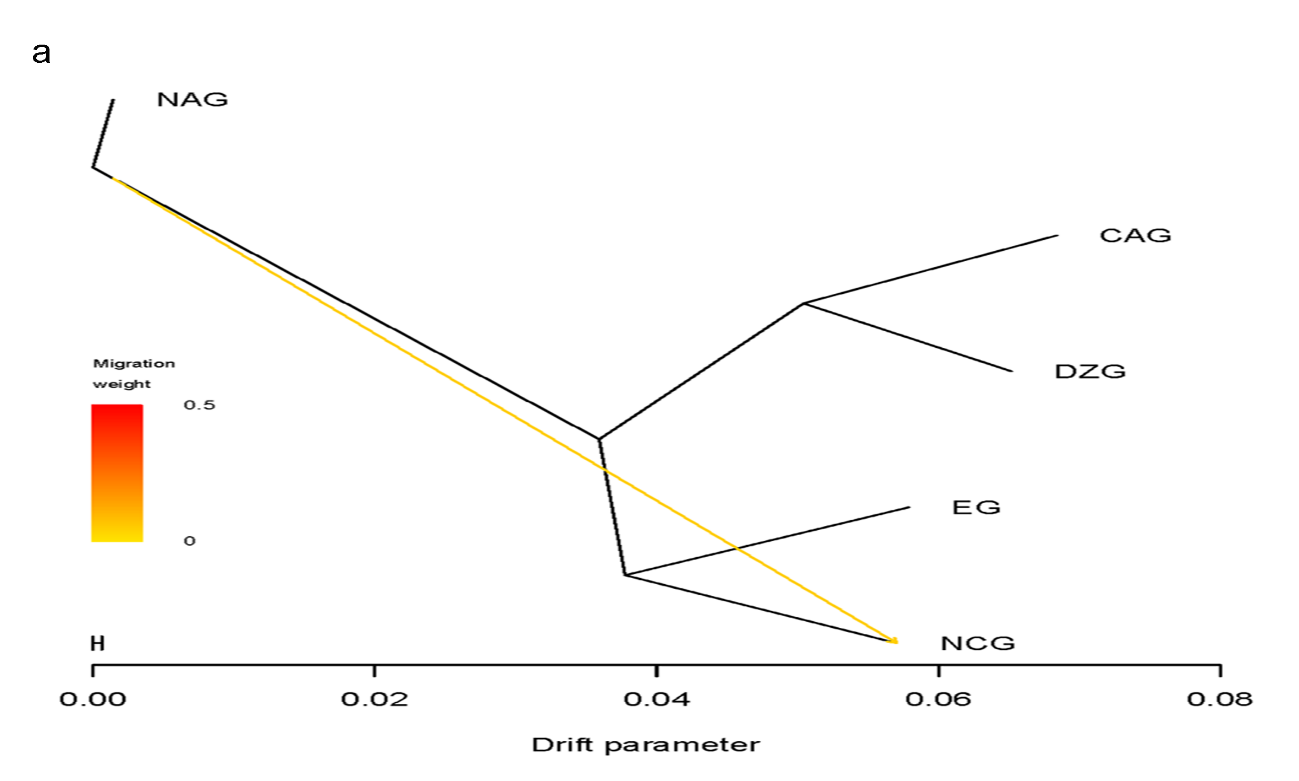

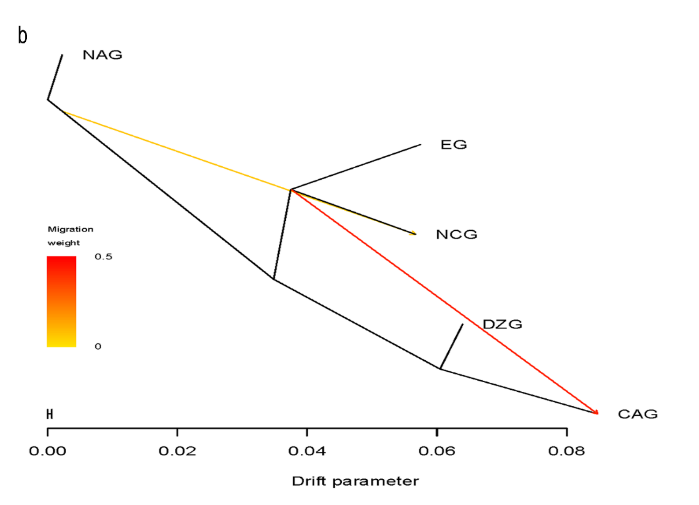

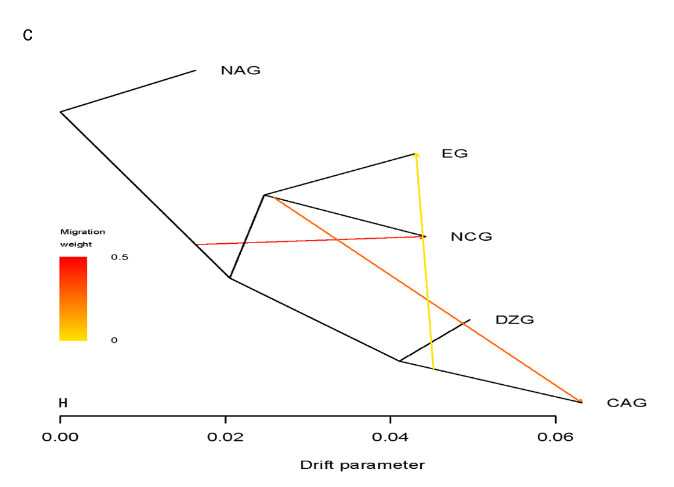


**Fig. 2 Detection of gene flow between five ecological groups accessions.** Lines represent gene flow; arrows indicate the direction of gene flow. The scale bar shows a tenfold average standard error of the entries in the sample covariance matrix. The color bar shows the migration weight: a red color denotes a strong gene flow, while a yellow color denotes a weak gene flow
